# Body-worn IMU array reveals effects of load on performance in an outdoor obstacle course

**DOI:** 10.1371/journal.pone.0214008

**Published:** 2019-03-21

**Authors:** Rachel V. Vitali, Stephen M. Cain, Lauro V. Ojeda, Michael V. Potter, Antonia M. Zaferiou, Steven P. Davidson, Megan E. Coyne, Clifford L. Hancock, Alyssa Mendoza, Leia A. Stirling, Noel C. Perkins

**Affiliations:** 1 Department of Mechanical Engineering, University of Michigan, Ann Arbor, Michigan, United States of America; 2 Department of Orthopedic Surgery, Rush University Medical Center, Chicago, Illinois, United States of America; 3 Combat Capabilities Development Command—Soldier Center, Natick, Massachusetts, United States of America; 4 Department of Aeronautics and Astronautics, Massachusetts Institute of Technology, Cambridge, Massachusetts, United States of America; 5 Institute for Medical Engineering & Science, Massachusetts Institute of Technology, Cambridge, Massachusetts, United States of America; University of Illinois at Urbana-Champaign, UNITED STATES

## Abstract

This study introduces a new method to understand how added load affects human performance across a broad range of athletic tasks (ten obstacles) embedded in an outdoor obstacle course. The method employs an array of wearable inertial measurement units (IMUs) to wirelessly record the movements of major body segments to derive obstacle-specific metrics of performance. The effects of load are demonstrated on (N = 22) participants who each complete the obstacle course under four conditions including unloaded (twice) and with loads of 15% and 30% of their body weight (a total of 88 trials across the group of participants). The IMU-derived performance metrics reveal marked degradations in performance with increasing load across eight of the ten obstacles. Overall, this study demonstrates the significant potential in using this wearable technology to evaluate human performance across multiple tasks and, simultaneously, the adverse effects of body-borne loads on performance. The study addresses a major need of military organizations worldwide that frequently employ standardized obstacle courses to understand how added loads influence warfighter performance. Importantly, the findings and conclusions drawn from IMU data would not be possible using traditional timing metrics used to evaluate task performance.

## Introduction

Body-borne loads have degraded human physical performance for millennia [1–3]. During Medieval times, steel plate body armor afforded protection in battle while simultaneously limiting mobility. In fact, modern experiments [2] reveal that Medieval body armor essentially doubles the energetic cost of locomotion (e.g., walking and running). This fundamental trade-off between increased capability (e.g. personal protection) versus physical performance (e.g. mobility and energy expenditure) remains pertinent today. Beyond degrading physical performance, body-borne loads may accelerate physical injury, fatigue, and other physiological changes [3]. Changes to metabolic, cardiopulmonary, and thermoregulatory processes may arise from the loads borne in diverse occupations including firefighting, law enforcement, search and rescue, and warfighting, as prime examples [4]. For instance, a current study relevant to law enforcement finds that stab resistant armor decreases performance during mobility, balance, and strength tasks [5]. In the context of the armor donned by warfighters, prior studies similarly confirm degradations in walking/marching performance (see, for example, [6–7]) and in running, rushing (rapid prone to sprinting back to prone), lifting, and many other tasks embedded in military obstacle courses [7–9]. The adverse effects of loads on marching performance in particular is well established and for body-borne loads of all kinds (e.g., armor, backpacks, hand-carried), collectively referred to as clothing and individual equipment (CIE).

Prior studies of marching performance reveal broad influences of added load as observed in both controlled (laboratory) conditions (e.g., via treadmills/force plates) and uncontrolled (outdoor, over ground) conditions (e.g., via course completion times). For instance, there is clear evidence of gait adaptation with added load [10–16] that is likely due to the need to increase stability and absorb larger ground reactions [10, 14]. Added load also increases walking energy expenditure [10, 12, 14] beyond that induced by increased grade alone [12] while also modifying knee biomechanics through reduced knee flexion/extension [16] and increased extension moment [11]. Incremental increases in backpack loads elicit proportional increases in both vertical and horizontal (anterior-posterior) ground reaction forces [13, 15], but without significant changes to the support phase of the gait cycle [15] for lower carried loads. These adaptations with load generally exhibit no gender differences [17] (when considering load relative to body mass), except possibly those caused by differing gender responses to backpack design [18]. Marching speed with loaded backpacks also depends on load distribution (front load versus rear load) and pack design (e.g., straps and webbing design, single versus double pack) [19–20], and the added loads also affect mood, comfort, and an array of physiological responses [21–22]. Beyond affecting marching performance, added load degrades performance in lifting [23], marksmanship [19–20, 23], and drop landings [24], all important additional tasks for warfighter performance.

Also pertinent to this paper are prior studies on the effects of body-borne loads in high-intensity tasks embedded in military obstacle courses. A review of previous studies [25] surveys the effects of added load across multiple obstacle course designs. In general, added load hinders tasks that induce large acceleration of the torso and/or limbs (e.g., start of locomotion, prone to sprinting, and lifting). For example, performance degradations arise in five tasks (25-yard sprint, standing long jump, agility run, reaction–movement test, and ladder climb) with male and female subjects carrying typical military loads (up to 37 kg) [26]. Studies exclusive to female subjects [27–28] demonstrate that absolute VO2 max (defined as oxygen uptake) and unloaded 3.2 km run time best predict the times needed to complete prescribed marches with added loads. Similarly, in the context of female subjects, studies [28–29] also explore possible correlations between performance under load (up to 25 kg) with predictor variables including the Army physical fitness test (APFT), treadmill VO2 sub max (defined as oxygen uptake), and anthropometric variables over six obstacles (low hurdles, agility run, low crawl, overload horizontal pipe, wall, and sprint).

While differing obstacle course designs reveal the influences of body-borne loads on physical performance, the need remains to standardize testing procedures for the systematic evaluation of load (i.e., CIE) on warfighter tasks. To this end, the US Marine Corp introduced the Load Effects Assessment Program (LEAP) which incorporates a standard (10-task) obstacle course that embeds combat-relevant movements and tasks. Physical performance in the LEAP obstacle course is quantified by the overall time to complete the course together with the times to complete each obstacle (measured using timing gates). A preliminary study [30] considers thirty-five subjects running the LEAP obstacle course under eight loading conditions (for a total of 280 course trials) and concludes that course and obstacle completion times, in general, exhibit significant variation with load. The reliability of the LEAP course completion time was specifically studied in [31], which demonstrates that completion times stabilize after four trials, and thus practice is required for consistent performance. The incorporation of marksmanship and communication tasks within the LEAP as well as additional measurement modalities are advocated in [32].

Although the times to complete an entire course or any obstacle within it are valid measures of performance, the times alone do not reveal the underlying biomechanical movements that further discriminate performance levels or reveal sub-movements that limit or enhance performance. Of course, measuring human movement (including critical sub-movements of torso and limbs) in the context of a large outdoor obstacle courses is largely precluded using standard video motion capture methods. However, new wearable technology in the form of miniature inertial measurement units (IMUs) offer an attractive answer to this challenge. Strapped directly to body segments, IMUs measure three-dimensional acceleration and angular velocity of those segments to inform biomechanical analyses. With that goal, prior studies deploy an array of IMUs attached to the major body segments and demonstrate how IMU-derived measurements reveal human performance across several obstacles featured in the LEAP course including the balance beam [33], agility run [34–35], vertical jump [36], vertical transfer (lifting) [37], stair case [38], and high crawl [39]. Collectively, these studies expose how the acceleration and angular velocity data harvested from select IMU/body segments define and discriminate performance levels specific to each obstacle and simultaneously reveal the critical sub-movements that drive performance. The objective of this study is to significantly expand this approach by deploying these newly developed methods to understand how performance (described and defined by IMU data) is modified by body-borne loads and in the context of a large LEAP-style obstacle course. We hypothesize that the performance on each obstacle is affected by load and that increased load results in degradations in performance as measured by those metrics.

We open with an overview of the experimental methods employed including the obstacle course design and the statistical methods used to understand the effects of load on performance. Results follow from performance metrics that are specific to the ten obstacles included in the course, and these are referred to as the sprint, casualty drag, vertical jump, window, balance beam, wall, agility run, bounding rush, high crawl, and vertical transfer obstacles. For each obstacle, we provide a brief description of how the obstacle was completed by the participants, an explanation of how performance is quantified via IMU data, the results of the statistical analyses, and a discussion of the effects of load on performance. Major findings are summarized in the conclusions.

## Materials and methods

### Participants and body-worn IMU array and other equipment

Twenty-two participants (15 male and 7 female, age 19.9 ± 2.0 yrs, height 1.78 ± 0.13 m, mass 78.7 ± 14.9 kg, mean ± SD) were recruited from a collegiate Reserve Officers’ Training Corps (ROTC) and club sports population. All participants self-reported inexperience with the obstacle course and were therefore considered novices. The University of Michigan Institutional Review Board approved the study and all participants gave informed consent. The individuals in this manuscript have given written informed consent (as outlined in PLOS consent form) to publish these case details.

**Fig 1:**
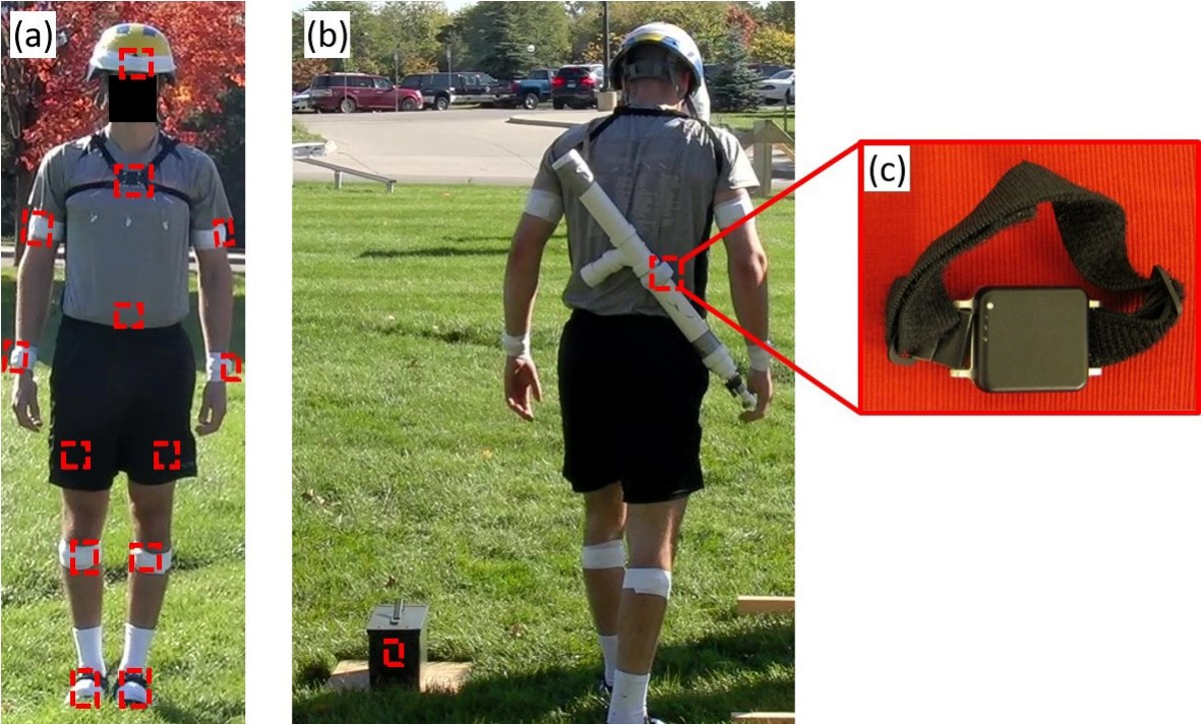
Participant outfitted with equipment (unloaded condition). (a) Participant wearing an array of IMUs (red rectangles) attached to major body segments and in the approximate locations shown (sacrum IMU on posterior). (b) Participant with the mock rifle slung over the shoulder with additional IMU nodes attached to the mock rifle and embedded in the ammo can. (c) Image of single IMU node with integrated Velcro strap.

Referring to Fig 1A, participants wore an array of 13 IMUs (Opal, APDM, Portland, OR, USA) attached (via Velcro straps and further secured by athletic tape) to major body segments including head, sternum, upper arms, forearms, sacrum, thighs, shanks, and feet. We deploy 13 IMUs in order to measure the kinematics of the 13 major body segments that may contribute to performance across all of the obstacles in the obstacle course. We further note that only a subset of the 13 IMUs are required to develop the obstacle-specific performance metrics for any one obstacle as described in the example studies [33–39] that are specific to only one obstacle. The IMU attached to the head was secured to a ballistic helmet. Participants also carried a mock (plastic) rifle (mass 3.2kg, length 0.75m) with an IMU mounted to the barrel (Fig 1B). Lastly, one IMU was embedded within an ammo can (constituting a 13.6 kg load) used in one obstacle (i.e., a manual lifting task; Fig 1B). Each IMU incorporates a triaxial accelerometer (6g range, 14-bit resolution, 650μg/√Hz noise floor) and a triaxial angular rate gyro (2000°/s range, 16-bit resolution, 0.03deg/sec/√Hz noise floor) sampled at 128Hz (Fig 1C). As further explained below, participants completed the obstacle course with and without carrying additional loads. For the loaded conditions, participants wore a (V-FORCE long) weight vest (Weightvest.com, Rexburg, ID, USA) laden with a select number of cast iron weights (mass 1.1kg, dimensions 10cm x 5cm x 4cm, equally distributed across front and back). It should be noted that the participant in Fig 1 represents the unloaded condition (no load vest).

### Obstacle course and testing protocol

**Fig 2:**
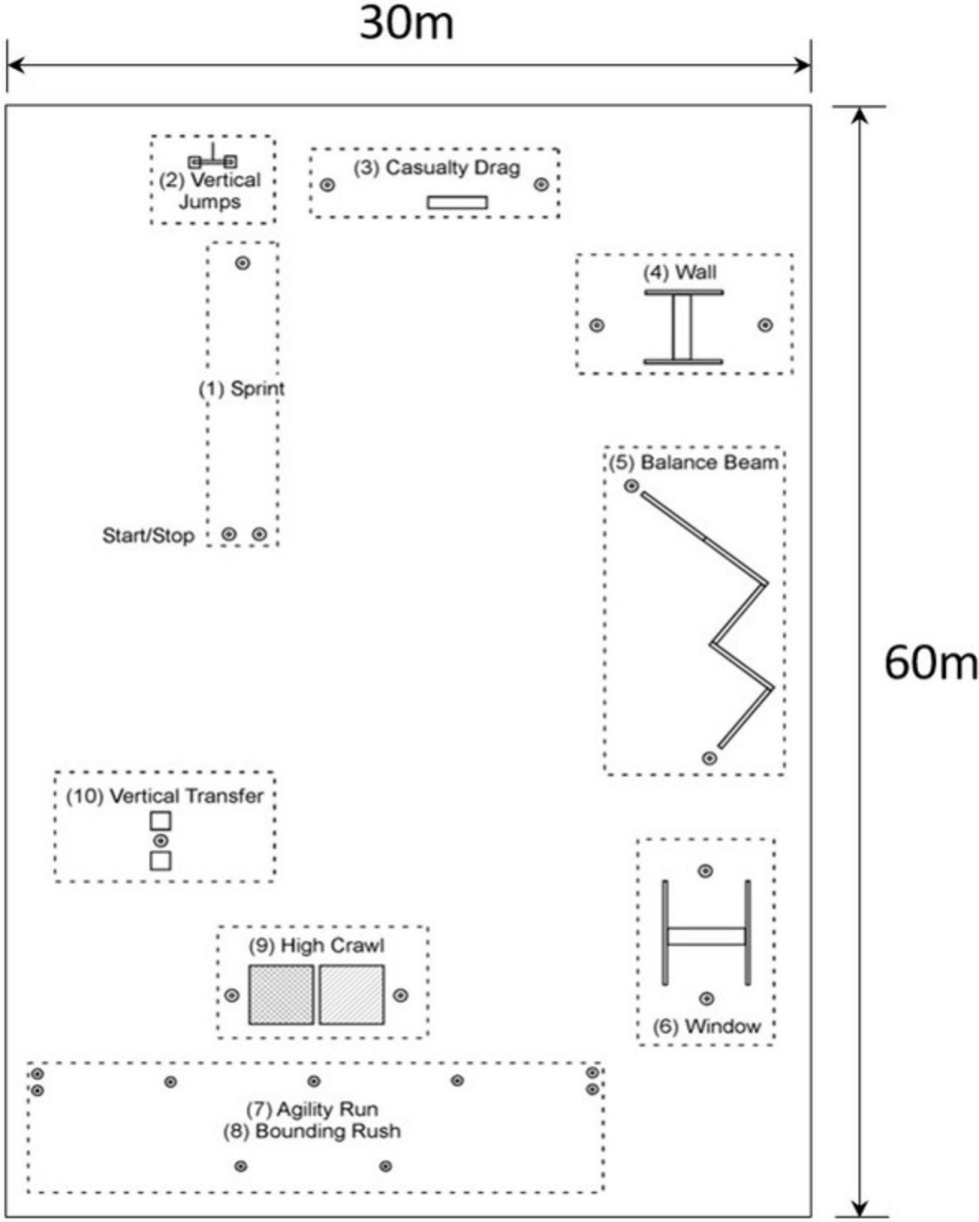
Outdoor obstacle course layout. Plan view of ten obstacles for a modified LEAP obstacle course [30–31].

The course consists of the ten obstacles identified in Fig 2 that form a subset of those found in the LEAP [30–31]. The ten obstacles, each described in greater detail in the Results and Discussion, require: 1) sprinting in a straight line, 2) jumping vertically, 3) dragging a load (a.k.a. casualty drag), 4) climbing over a wall, 5) traversing a balance beam, 6) climbing through a window opening, 7) running an agility course, 8) alternating running and prone target acquisition in the agility course (a.k.a. bounding rush), 9) crawling on knees and elbows/forearms (a.k.a. high crawl), and 10) repeatedly lifting a load onto a raised platform (a.k.a. vertical transfer).

Participants completed the obstacle course four times: two times without added load (unloaded condition: UL), once wearing an additional 15% of their body weight (loaded condition: 15BW), and once wearing an additional 30% of their body weight (loaded condition: 30BW). They did so over two testing sessions (on separate days) each of which included the UL condition and one of the two loaded conditions with 15 minutes in between for recovery. The order of loading conditions within and between testing sessions was randomized. For context, descriptive statistics for the additional loads are reported in Table 1. Note that the mean load representing 30% body weight (23.6 kg) is comparable to typical infantry fighting loads.

**Table 1:** Minimum, mean, maximum, and standard deviation (STD) of additional loads (in kg) secured to the participants.

	Min (kg)	Mean (kg)	Max (kg)	STD (kg)
**15BW**	8.3	11.8	16.9	2.2
**30BW**	16.6	23.6	33.8	4.4

Prior to testing, each participant walked through the course and received instruction on how to complete each obstacle. Simultaneously, they were encouraged to try each obstacle to familiarize themselves with the required movements and to reduce learning effects [31]. Participants repeated this training during the first loaded condition to experience how the load may affect movement. It should be noted that while participants were instructed to use maximal effort to completing all obstacles, heart rate and metabolic consumption were not recorded to assess whether that was the case. However, the randomization of loading condition was used to control for fatigue effects.

At the beginning of each testing session, participants first completed a sequence of calibration movements that included: 1) quiet standing, 2) toe touching, 3) standing on one leg and executing a pedaling motion with the contralateral leg (and then repeated for opposite leg condition), and 4) quiet standing. Each movement lasted approximately 10 seconds. The IMU data from this sequence of calibration movements was later used to estimate anatomical axes of major limb segments for specific obstacles noted in the Results and Discussion. Participants were then instructed to complete each of the ten obstacles as quickly as possible with very short periods (2–5 seconds) of quiet standing just prior to and just after each obstacle. The periods of quiet standing serve to 1) further identify the start and end of each activity, and 2) establish rest initial conditions for subsequent integration of IMU data (and drift error reduction) for obstacle-specific performance analyses (refer to Results and Discussion). A trained member of the research team used a binary switch, which was synchronized with the IMU data, to mark the beginning and end of each obstacle and the period of quiet standing.

### Data analysis

Data analysis begins with parsing the data stored from the IMUs over the entire obstacle course into ten smaller data segments, one segment for each of the ten obstacles. The obstacle-specific IMU data represents the input to each of the obstacle-specific performance analyses described in the Results and Discussion (and the references cited therein). The performance analyses yield metrics of performance specialized to each obstacle for studying the effects of added load on performance. The analyses were conducted with a custom software interface that enabled parsing data, aggregating demographic data, and batch processing data files.

#### Parsing IMU data for each obstacle

Using the signal from the binary switch that was used to mark the beginning and end of each obstacle, the overall obstacle course data sets were parsed into smaller obstacle-specific data sets. The obstacle-specific data sets are input to obstacle-specific algorithms that yield obstacle-specific metrics of performance. These obstacle-specific algorithms are summarized in the Results and Discussion section so that the reader can better understand the IMU-derived performance metrics in the immediate context of each obstacle where the associated results are presented and discussed.

#### Statistical analysis of load effects on performance

We hypothesize that the obstacle-specific metrics of performance are affected by load and that increased load results in degradations in performance as measured by those metrics. Since participants complete the obstacle course in all loading conditions (unloaded (UL), carrying 15% body weight (15BW), and carrying 30% body weight (30BW)), a repeated measures analysis of variance (ANOVA) framework was used to evaluate the effects of load on performance. Several participants were eliminated from consideration for specific obstacles whenever: 1) the participant failed to complete all loading conditions, 2) the data for one or more sensors was lost (sensor failure), or 3) the participant did not follow instructions. Each obstacle-specific algorithm is reliant on basic instructions about how each obstacle is performed and violations of these instructions (e.g. a subject not beginning and ending an obstacle in quiet standing or a subject not sprinting at maximum effort) renders the data inaccurate for subsequent analysis.

For each performance metric, the residuals were evaluated for normality with Shapiro-Wilk normality tests and q-q plots. If a significant deviation from the normal distribution was present, an appropriate data transformation was performed to correct the type of skewness present in the residual distribution, after which the ANOVA was conducted again on the transformed data. The residuals were also checked for heteroscedasticity with Mauchly’s test for sphericity. If the sphericity assumption is violated, the F-statistic from the ANOVA was evaluated with adjusted degrees of freedom via a Greenhouse-Geiser correction. Additionally, effect sizes (*η*^2^) were calculated to quantify the magnitude of the effect that load has on the performance metric. Post-hoc analyses via Tukey pairwise-comparisons were conducted for performance metrics with significant F-statistics. For each comparison, effect sizes (Cohen’s *d*) were also calculated to evaluate the magnitudes of the differences between loading conditions. All statistical tests were evaluated at a significance level α = 0.05. The relative magnitudes (as defined in [40]) of the effect sizes for the ANOVA and the Tukey post hoc analyses are summarized in Table 2.

**Table 2:** Relative magnitudes for the effect sizes for the ANOVA ($\eta^{2}$) and Tukey (*d*) analyses [40].

Effect Size	*η*^2^ ANOVA	*d* Tukey
**Small**	0.01	0.2
**Medium**	0.06	0.5
**Large**	0.14	0.8

## Results and discussion

In the context of presenting results, we also provide a brief summary of the design of each obstacle, an overview of the obstacle-specific performance metrics, and a discussion of salient results illustrating the effect of load on performance for each obstacle. We reference the comprehensive results from the repeated-measures ANOVA and Tukey post hoc analyses reported in the Supplemental Information. Each obstacle is presented in the numerical order illustrated in Fig 2, beginning with the sprint obstacle. A comprehensive summary of all results from all obstacles is illustrated and discussed in the Summary and Conclusions section.

### Sprint

This obstacle is an 18.3 m (60 ft) straight sprint on level terrain between pairs of start and finish cones. Participants were instructed to stand still in a ready position at the start cones until instructed to sprint. They completed a maximal effort sprint with the rifle in ready position (i.e. both hands holding the mock rifle in front with barrel down) through the finish cones before decelerating and returning to rest.

Data from the sacrum IMU was used to estimate the instantaneous horizontal body speed following a modification of the procedure employed in [34] as briefly summarized here. The start and end of the sprint were identified when the sacrum acceleration magnitude dropped below 10% of the maximum acceleration magnitude during the trial. The quaternion (IMU orientation) data (output from Motion Studio, APDM, Portland, OR, USA) were employed to resolve the sacrum acceleration in a world frame defined by mutually orthogonal vertical and horizontal directions. Integrating the resulting (world frame) acceleration components (with zero velocity initial condition) yields the instantaneous sacrum (body) velocity throughout the sprint. Integration drift error was estimated and removed by computing linearly-varying corrections to all three velocity components that enforce zero velocity at the end of the trial when the participant returned to being still [34]. The resulting (world frame) velocity components were then integrated to estimate the instantaneous position of the sacrum throughout the sprint. The position coordinates reveal the overall heading direction, which is used to compute the instantaneous horizontal speed *V*(*t*) in the direction of sprinting.

Furusawa, et al. [41] observed that the horizontal body speed *V*(*t*) during a maximal effort sprint (without fatigue) obeys

$$V\left(t\right)=V_{t}\left(1-e^{-\frac{t}{\alpha }}\right)$$ (1)

Where *V*(*t*) is the maximum speed the participant can attain and *α* is the characteristic time of the exponential rise. The two parameters (maximum speed *V*(*t*) and characteristic time *α*) were estimated for each trial using standard curve fitting to the horizontal speed *V*(*t*) sampled from the start of the sprint to the time of maximum *V*(*t*). An illustrative example of the horizontal body speed with the fitted curve is provided in Fig A in S1 File. Additionally, the estimated maximum acceleration *a*_*max* =_ *V*_*t*_/*α* follows from (1). Integration of (1) enables solution of the sprint time *T*_*sprint*_ from

$$L=V_{t}T_{sprint}+V_{t}\alpha \left(e^{-\frac{T_{sprint}}{\alpha }}-1\right)$$ (2)

Where *L* is the known sprint length (18.3 m). Collectively, four sprint performance metrics (*V*(*t*), α, *a*_*max*_,*T*_*sprint*_) represent the input to the aforementioned statistical analysis. The results of the ANOVA and Tukey post hoc analyses are reported in Table A in S1 File. Significant results are presented and discussed below.

The ANOVA reveals load has a significant effect on three performance metrics, namely estimated sprint time *T*_*sprint*_ (F(2,20) = 17.2, p<0.001, η^2^ = 0.63), maximum speed *V*_*t*_ (F(2,20) = 14.4, p<0.001, η^2^ = 0.59), and maximum acceleration *a*_*max*_ (F(2,20) = 14.9, p<0.001, η^2^ = 0.60). No relationship with load was observed for the characteristic time *α*.

**Fig 3:**
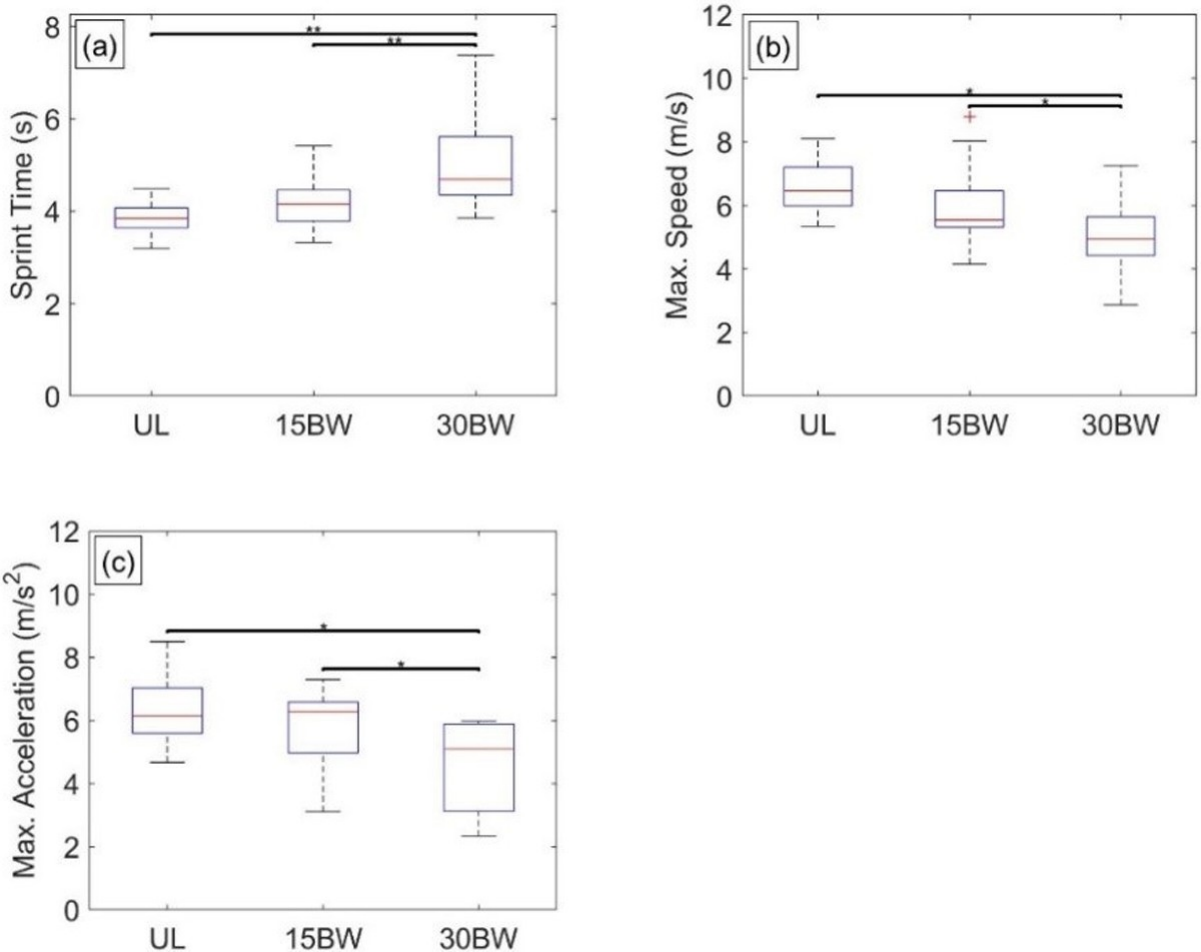
Sprint statistical results. Boxplots depicting the results from the Tukey post hoc analysis for a) sprint time, b) maximum speed, and c) maximum acceleration. The bars denote significant differences between loading conditions at a significance level α = 0.05*, 0.01**, 0.001***.

Fig 3 illustrates the significant results of the Tukey post hoc analysis. First, observe that sprint time increases with increased load as expected (Fig 3A). No significant difference was observed in the UL-15BW (p = 0.07, *d* = -0.62) comparison while significant differences and large effect sizes are observed for UL-30BW (p<0.01, *d* = -1.54) and 15BW-30BW (p<0.01, *d* = -1.00) comparisons. These results suggest a significant, but potentially nonlinear increase in sprint time with increased load.

The maximum speed (Fig 3B) is reduced with increased load. No significant difference was found in the UL-15BW comparison (p = 0.17, *d* = 0.52) whereas significant differences (and large effect sizes) were found for the 15BW-30BW (p = 0.03, *d* = 0.83) and UL-30BW (p<0.001, *d* = 1.63) comparisons. As with sprint time, this suggests a significant, potentially nonlinear decrease in maximum speed with increased load. Finally, and also expected, maximum acceleration (Fig 3C) decreased with increased load. The 15BW-30BW (p<0.01, *d* = 0.90) and UL-30BW (p<0.01, *d* = 1.31) comparisons have significant differences (and large effect sizes), which suggests a possibly nonlinear decrease in maximum acceleration with increased load. Collectively, these results suggest that there may be a tradeoff wherein added load may partly aid participants (by increasing their horizontal ground reaction force) before significantly penalizing them for overall increased mass.

### Vertical Jumps

The vertical jump obstacle requires participants to complete three maximum effort countermovement vertical jumps. During countermovement jumps, participants start in an upright standing position, then they flex their knees and hips to lower their center of mass (the countermovement phase), and finally they propel themselves largely upwards by rapidly extending their joints (propulsion phase). A vertical jump stand (Vertec; Sports Imports, Hilliard, OH) is used to motivate participants to jump as high as possible. They also assume a quiet standing posture before and after completing each jump. Participants did not carry the mock rifle during this obstacle.

Performance in the vertical jump is quantified by metrics obtained with data measured from the sacrum-mounted IMU [36]. The IMU-derived sacral kinematics (vertical acceleration, vertical velocity, and vertical position) are used to define eight performance metrics. First, each jump was divided into a countermovement phase (from initiation of the countermovement to the bottom of countermovement which is defined by the maximum downward vertical displacement) and a propulsion phase (from the bottom of the countermovement to the takeoff which is identified by the maximum vertical velocity). An illustrative example of these phase definitions is provided in Fig B in S1 File. For the countermovement phase, we define: 1) countermovement duration as total time of this phase, 2) countermovement velocity as the average vertical velocity during this phase, and 3) countermovement depth as the maximum downward vertical displacement at the end of this phase. For the propulsion phase, we define: 4) propulsion phase duration as the total time of this phase, 5) propulsion phase acceleration as the average acceleration during this phase, and 6) takeoff power as the average power during the positive power region of the propulsive phase. At the end of the propulsion phase, we define 7) takeoff velocity as the velocity at the end of the phase. Finally, using projectile motion equations we estimate the 8) jump height as the maximum height achieved given the takeoff velocity. These metrics are averaged across all three jumps to yield mean values. The results of the ANOVA and Tukey post hoc analyses for the vertical jump are reported in Table B in S1 File. Salient results are presented and discussed below.

The ANOVA reveals that load is significantly related to overall jump height (F(1.6,28.6) = 17.0, p<0.001, *η*^2^ = 0.49), countermovement velocity (F(1.5,27.7) = 20.4, p<0.001, *η*^2^ = 0.53), countermovement depth (F(2,36) = 8.2, p = 0.001, *η*^2^ = 0.31), propulsion phase acceleration (F(1.3,24.3), p<0.001, *η*^2^ = 0.65) and takeoff velocity (F(1.3,22.8) = 63.0, p<0.001, *η*^2^ = 0.78). No relationships with load were observed for countermovement duration, propulsion phase duration, or takeoff power.

**Fig 4:**
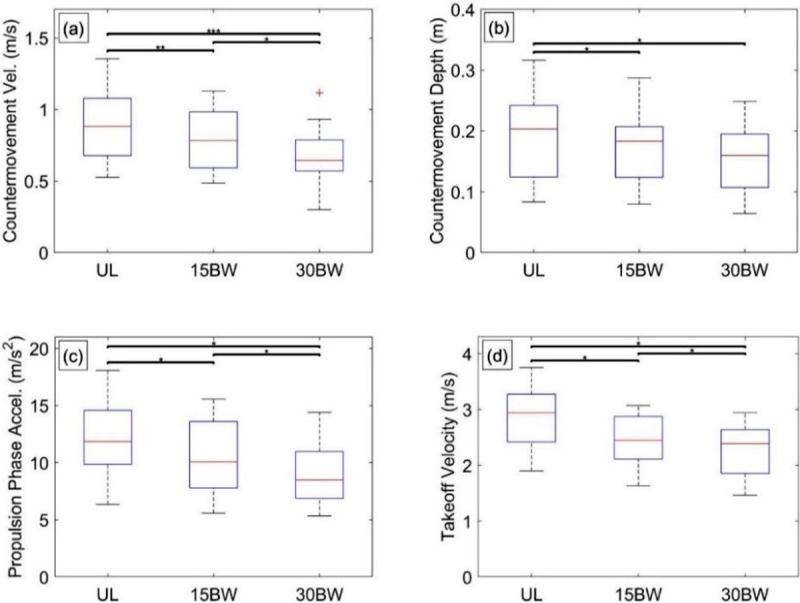
Vertical jump statistical results. Boxplots depicting the results from the Tukey post hoc analysis of vertical jump metrics for a) jump countermovement velocity, b) jump countermovement depth, c) jump propulsion phase acceleration, and d) jump takeoff velocity. The bars denote significant differences between loading conditions at a significance level α = 0.05*, 0.01**, 0.001***.

Fig 4 illustrates selected results from the Tukey post hoc analyses. Consistent with previous studies [36, 42], vertical jump performance is significantly affected by added load. To start, the three pairwise comparisons for countermovement velocity are significant, indicating participants decrease countermovement velocity when carrying added load (Fig 4A). Only the UL-15BW (p = 0.01, *d* = 0.34) and UL-30BW (p<0.01, *d* = 0.59) comparisons were significant for countermovement depth (Fig 4B). Like [36], the countermovement velocity decreases with load and this change is largely driven by decreasing the countermovement depth with load, as load does not have a significant relationship with countermovement duration.

Furthermore, the three pairwise comparisons for propulsion phase acceleration are all significant indicating that, as anticipated from Newton’s second law, added load results in decreased acceleration during the propulsion phase (Fig 4C). Consequently, and also as expected, the three pairwise comparisons for takeoff velocity (Fig 4D) are significant, though the effect sizes between the UL-15BW (p<0.001, *d* = 0.78) and 15BW-30BW (p = 0.001, *d* = 0.43) comparisons indicate the decreases are likely nonlinear with increasing load. Assuming largely upward projectile motion after the participant is airborne, jump height is proportional to the square of takeoff velocity. Thus, the decrease in jump height is mainly explained by the decrease in takeoff velocity. We again note that propulsive power showed no significant dependence on load unlike the take-off velocity that does exhibit significant load dependence. Consequently, participants must significantly increase their muscle activation (not measured) with load in order to maintain the near-constant power (measured) under increasing load.

### Casualty drag

**Fig 5:**
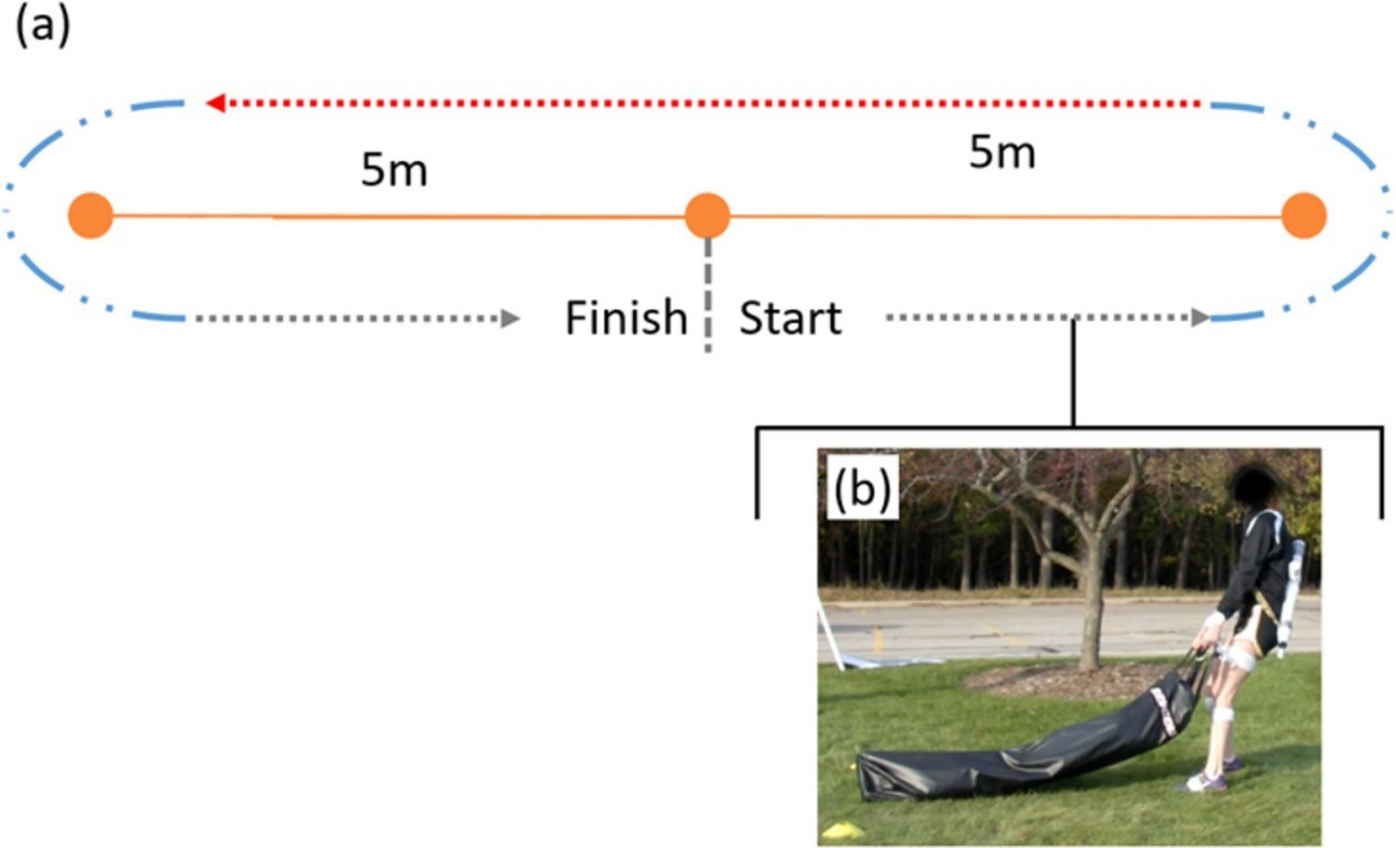
Casualty drag obstacle. a) The layout for the casualty drag obstacle defined by two cones separated by 10m. Participants drag a heavy load (bag) in a loop around the cones as shown by the dashed lines. Blue and red sections of the path indicate turn and straightaway phases, respectively. b) A photo illustrating a participant dragging the load near the start.

The casualty drag obstacle requires participants to partially lift and drag a large load (82 kg bag) on the ground while walking a prescribed course; refer to Fig 5. The course is marked by two cones separated by 10 meters. With the mock rifle slung across their back (illustrated in Fig 5B below), participants drag the load in a loop around the cones, starting and ending the loop approximately half way between the cones as illustrated.

Performance in the casualty drag is deduced from four metrics relating to participant speed, derived from the estimated trajectories of each foot following [43]. The foot trajectories are decomposed into turn phases and straight phases. The turn phases are portions of the foot trajectory within one meter of the trajectory extrema (i.e., the portion of the trajectories nearest the cones) and the straightaway phases lie between the turn phases; refer to Fig 5A. The start and end of the casualty drag are determined by the first and last observable strides (i.e. the first and last stride lengths exceeding 50% of the maximum stride length). The horizontal components of each foot trajectory are smoothed using a cubic spline function and instantaneous foot velocities are computed from the smoothed trajectories. Finally, the average of the left and right foot velocities yields an estimate of the instantaneous body speed following [35].

From these computations, we report four speed-related performance metrics; namely, 1) average body speed, 2) average turn body speed, 3) average straightaway body speed, and 4) obstacle time (time of casualty drag). The average body speed follows from the average of the instantaneous body speed across the obstacle time. The average turn speed and the average straightaway body speed follow from the average instantaneous body speeds during the two turn phases and during the back 10-meter straightaway, respectively. These four performance metrics are used in the aforementioned statistical analysis. The results of the ANOVA and Tukey post hoc analyses for the casualty drag are reported in Table C in S1 File. Significant results are presented and discussed below.

The ANOVA reveals relationships with load for all four metrics: average body speed (F(2,24) = 7.8, p<0.01, η^2^ = 0.39), average turn body speed (F(2,24) = 5.8, p<0.01, η^2^ = 0.33), average straightaway body speed (F(2,24) = 5.7, p<0.01, η^2^ = 0.32), and obstacle time (F(1.16,13.9) = 4.9, p = 0.04, η^2^ = 0.29).

**Fig 6:**
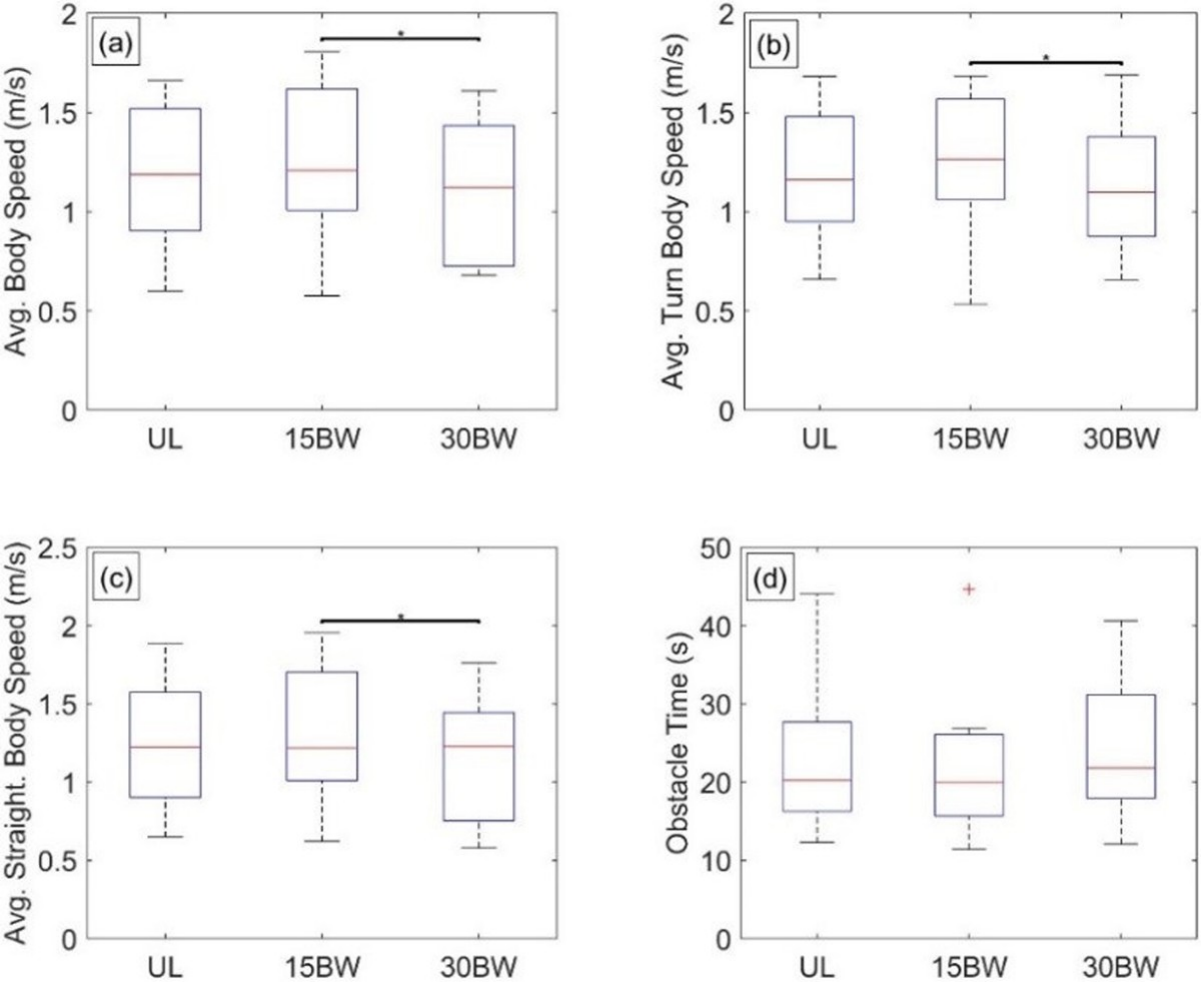
Casualty drag statistical results. Boxplots depicting the results from the Tukey post hoc analysis for a) average body speed, b) average turn body speed, c) average straightaway body speed, and d) obstacle time.

The results of the Tukey post hoc analyses for the metrics are illustrated in Fig 6. Inspection of Fig 6A–6C reveals that only the BW15-BW30 comparisons are significant for the indicated metrics (a: p = 0.02, *d* = 0.39; b: p = 0.04, *d* = 0.43; c: p = 0.04, *d* = 0.40) while no significant differences with load arises for obstacle time (Fig 6D). Thus, degradation in speed performance is likely nonlinear or even absent. A possible explanation is that load added to the torso may partially aid as much as it hinders dragging performance. Consider that dragging requires significant horizontal ground reaction forces and that these forces are limited by the available friction (hence also vertical ground reaction) between the terrain and the participants’ shoes. Adding load to the torso increases the friction limit (via increased vertical ground reaction) thereby possibly making the task easier. If a subject’s weight is small relative to the load, some additional weight may promote greater leverage against the load making the task easier. However, adding significant load to the torso also increases the overall load that the upper body must support leading to the observable pauses some participants employed as a result of fatigue, readjusting their grip, cutting inside the cones, and readjusting their path. Such added pauses immediately impact all speed-related performance metrics.

In summary, the differences observed in the Tukey post hoc analyses are largely non-monotonic in that added load is associated with improved as well as degraded performance depending on the load level.

### Window

The window obstacle is a freestanding wooden wall with an (0.91 m X 0.91 m) opening (1.22 m) off of the ground. Participants are instructed to quickly approach the window and use any technique they choose to pass through the window opening as quickly as possible, only using the window sill/frame for leverage. Participants begin the obstacle in a quiet standing posture (4.5 m) in front of the window. After passing through the window opening, participants run and stop (approximately 4.5 m past the window) returning to quiet standing posture. While passing through the window, participants were required to maintain possession of the mock rifle with at least one hand at all times.

**Fig 7:**
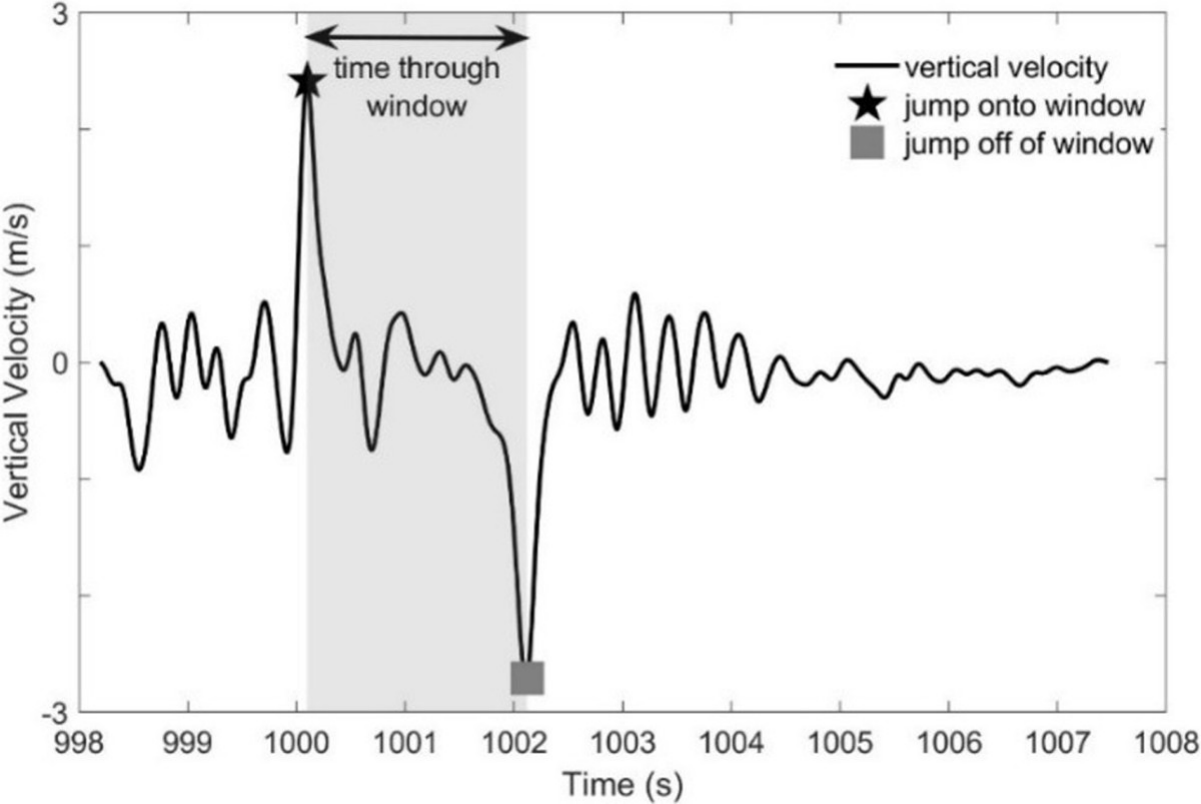
Drift-corrected vertical velocity of the sacrum IMU for a sample trial. Maximum vertical velocity (black star) corresponds to the participant jumping up onto the window. Minimum vertical velocity (gray square) corresponds to the participant jumping down off of the window. Time through the window is the interval between these points.

Performance in the window obstacle is quantified by metrics obtained with data from the sacrum-mounted IMU. Metrics were only calculated from successful attempts. We calculate sacral kinematics (acceleration, velocity, and position) using a zero velocity update algorithm [33, 43] with participants being still (zero velocity) both before beginning the obstacle and just after completing the obstacle. From the sacral kinematics, we define the following performance metrics: 1) time to pass through the window opening, 2) horizontal approach velocity, 3) vertical takeoff velocity, 4) vertical takeoff power, and 5) vertical landing velocity. The time to pass through the window opening is revealed by the vertical sacrum-velocity (Fig 7). A large positive peak indicates the movement up from the ground onto the window sill, whereas a large negative peak indicates the jump or drop down from the window sill onto the ground. The difference between these times is the time required to pass through the window opening.

The horizontal approach velocity is the maximum horizontal velocity prior to the movement up to the window sill. The vertical takeoff velocity is the maximum vertical velocity during the movement up from the ground onto the window sill. The vertical takeoff power is the average power in the vertical direction during the takeoff phase (movement up to the window sill), where power is calculated from the vertical acceleration, vertical velocity, and mass of the participant plus the mass of the added load. The vertical landing velocity is the peak vertical velocity in the downward direction during the drop from the window sill to the ground. The results of the ANOVA and Tukey post hoc analyses for the window obstacle are reported in Table D in S1 File. Significant results are presented and discussed below.

The ANOVA reveals that load has a significant relationship with the time to pass through the window opening (F(1.0,18.7) = 6.4, p = 0.020, *η*^2^ = 0.26), horizontal approach velocity (F(2,36) = 5.6, p = 0.007, *η*^2^ = 0.24) and vertical takeoff velocity (F(1.5,27.5) = 7.3, p = 0.005, *η*^2^ = 0.29). Vertical takeoff power and vertical landing velocity did not exhibit significant relationships with load. Thus, the vertical takeoff power for the window exhibits the same trend with load as that for the vertical jump.

**Fig 8:**
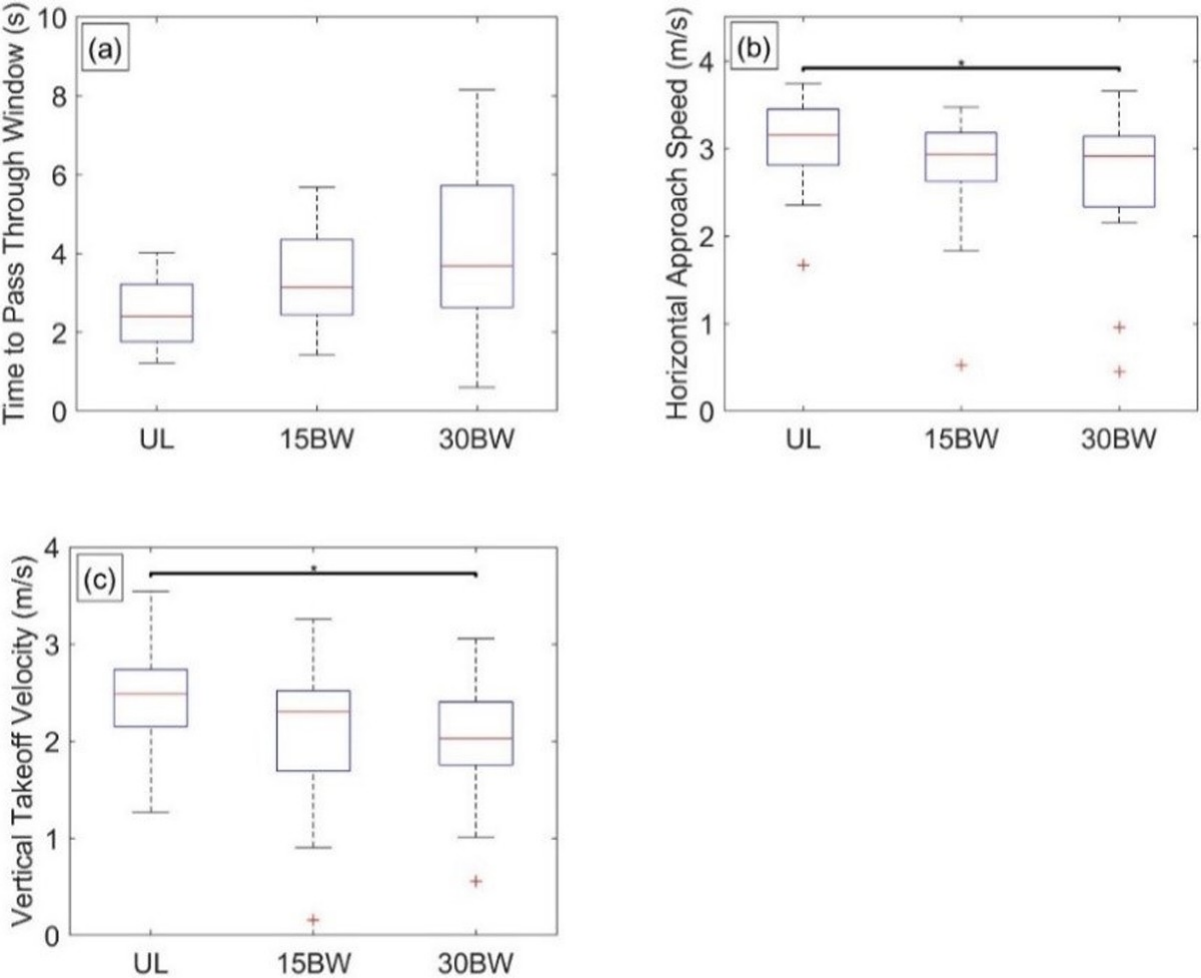
Window statistical results. Boxplots depicting the results from the Tukey post hoc analysis of window obstacle metrics for a) time to pass through the window opening, b) horizontal approach velocity, and c) vertical takeoff velocity. The bars denote significant differences between loading conditions at a significance level α = 0.05*, 0.01**, 0.001***.

Fig 8 illustrates selected results from the Tukey post hoc analyses. Despite a significant relationship to load, there are no significant pairwise comparisons between load conditions in the time to pass through the window opening (Fig 8A). While effect sizes for added load are consistent with increased time to pass through the window opening (UL-15BW: d = -0.68, UL-30BW: d = -0.75), differences between load conditions are not significantly different.

Only the UL-30BW comparison is significant for horizontal approach velocity (p<0.01, *d* = 0.59; Fig 8B) and vertical takeoff velocity (p<0.01, *d* = 0.71; Fig 8C). Participants approach the window more slowly when carrying additional load. Similar to the vertical jump results, takeoff velocity decreases with added load. Our results suggest that participants exert their maximum power (constant effort) in all load conditions. With increased mass due to the added load, the same (maximum) power results in decreased acceleration during the propulsive phase of the jump and thus decreased vertical takeoff velocity.

### Balance beam

**Fig 9:**
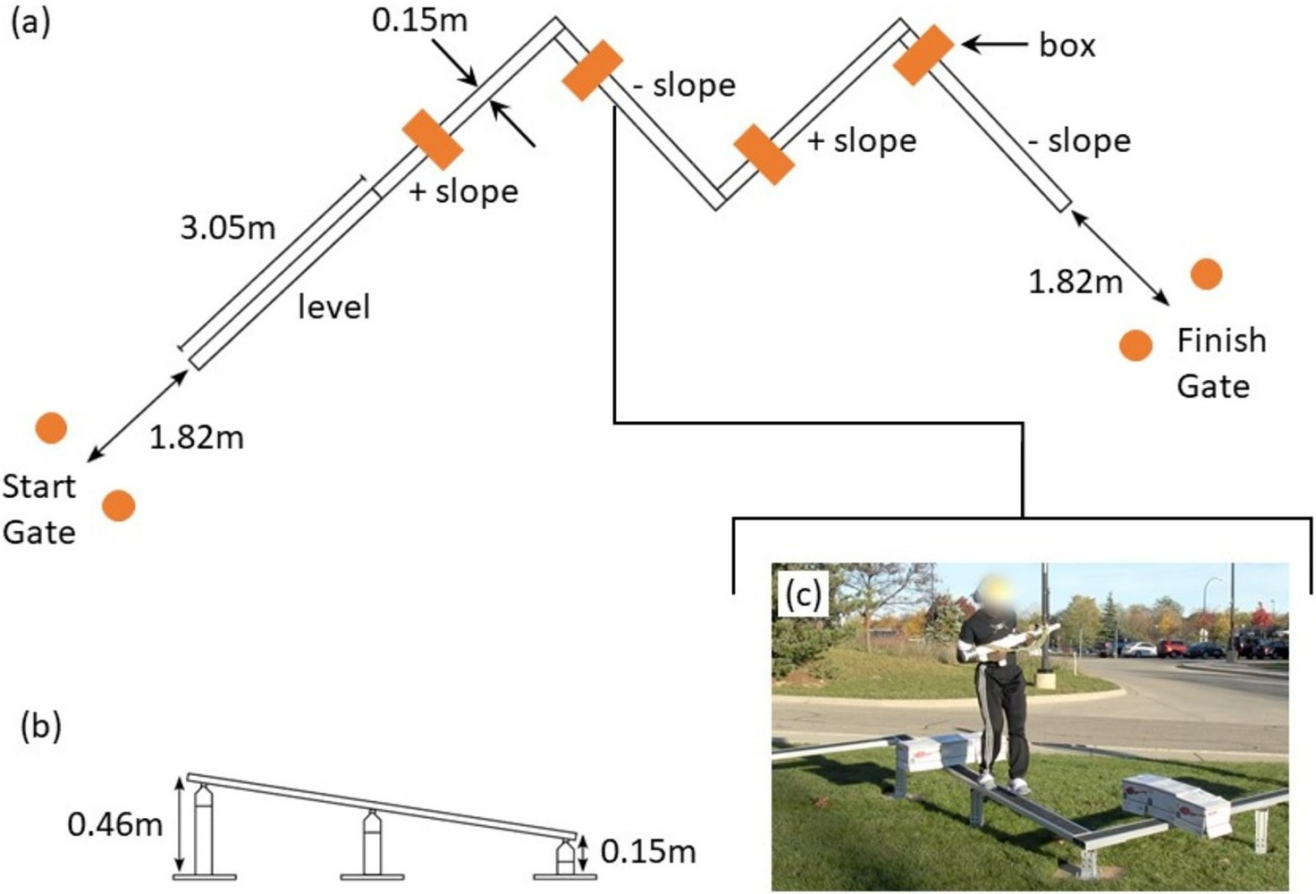
Balance beam obstacle. The layout for the balance beam obstacle in both (a) a top-down view and (b) a side view (b). (c) A photo of a participant completing the obstacle. The balance beam obstacle is composed of five elevated aluminum planks (0.15 m wide, 3.05 m long). The first plank is level, whereas the other planks alternately slope up or down by 9 degrees. The junction between the first and second planks is straight, whereas the other junctions are alternating right and left 90 degree turns. Four boxes (0.20 X 0.20 X 0.76 m) are placed on the beam to create an additional challenge for participants. The boxes are placed 1.04 m, 1.02 m, 0.71 m, and 0.30 m from the leading edge of the second, third, fourth and fifth planks, respectively.

The balance beam obstacle requires participants to traverse a balance beam as quickly as possible without stepping or falling off of the beam and maintaining control of the mock rifle with both hands in ready position. The balance beam obstacle (Fig 9) is composed of five elevated aluminum planks. Boxes, four in total, are placed on the beam to create an additional challenge for participants. The design of the balance beam obstacle incorporates the major features of the balance beam obstacle in the aforementioned LEAP [30–31].

Performance in the balance beam obstacle is quantified by metrics obtained with data from four of the body worn IMUs (two feet, sacrum, and sternum). The performance metrics are detailed in [33] and a brief summary is provided here. The IMU-derived performance metrics are: 1) time to traverse the beam, 2) average turn time, 3) average box step over time, 4) mean step time, 5) standard deviation of step time, 6) percentage of time spent in double support, and 7) ratio of root-mean squared (RMS) medial-lateral (M-L) acceleration to anterior-posterior (A-P) acceleration. Similar to the window obstacle, the time to traverse the beam is revealed by the vertical sacrum-velocity; a large positive peak indicates the step up from the ground onto the balance beam, whereas a large negative peak indicates the step down from the beam onto the ground. Using foot trajectories calculated from foot-mounted IMUs [43], we identify when participants turn (when directions of two successive strides differ significantly) and when participants step over an obstacle (large stride height). Turn time for a turn is defined by the time between the start of the first stride to initiate the turn (first stride in the new direction) and the end of the last stride to finish the turn (last stride in the new direction); average turn time is the average of the times of all three turns. Similarly, the box step over time is the time between the start of the first stride over a box and the last stride over a box; average box step over time is the average of times from all four boxes. Mean and standard deviation of the step time is the mean and standard deviation of all step times (time between consecutive foot strikes). Foot strike and push-off times define the phases of gait, which enable calculation of the total time spend in double support. Balance corrections may manifest as rapid lateral movements of the body in order to reposition the center of mass above the base of support. The ratio of RMS M-L sacrum acceleration to that of the A-P acceleration measures the magnitude of the M-L balance corrections relative to the A-P acceleration needed to negotiate the balance beam at high speed [33]. The results of the ANOVA and Tukey post hoc analyses for the balance beam are reported in Table E in S1 File. Salient results are presented and discussed below.

The ANOVA reveals load has significant relationships with the time to traverse the beam (F(2,30) = 20.6, p<0.001, *η*^2^ = 0.58), average box step over time (F(1.32,19.7) = 7.4, p<0.01, *η*^2^ = 0.33), mean step time (F(2,30) = 10.9, p<0.001, *η*^2^ = 0.42), standard deviation of step time (F(2,30) = 4.0, p = 0.03, *η*^2^ = 0.21), and the ratio of RMS M-L acceleration to A-P acceleration (F(1.29,19.3) = 15.7, p<0.001, *η*^2^ = 0.51). No relationships with load were observed for average turn time or percentage of time spent in double support.

**Fig 10:**
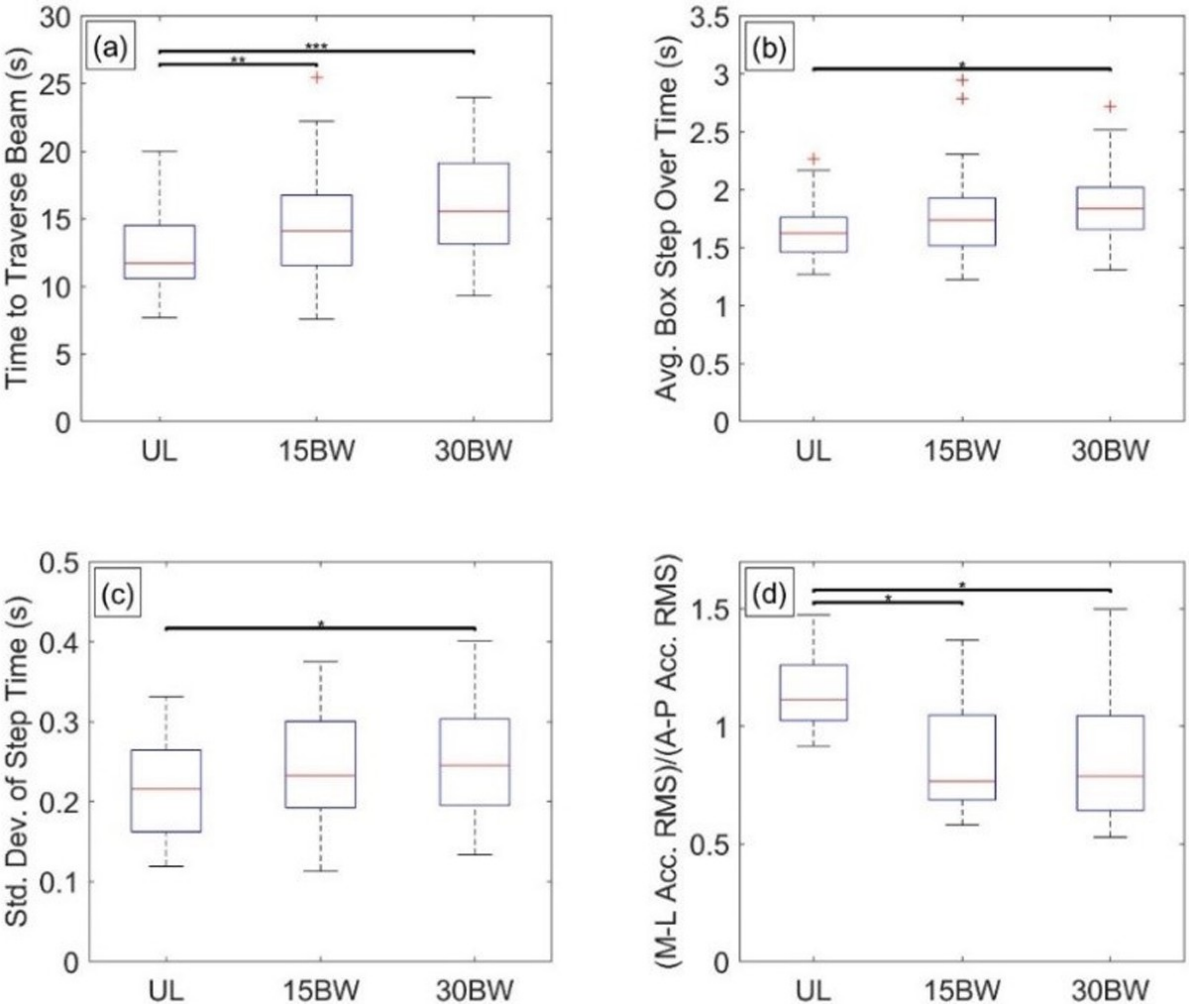
Balance beam statistical results. Boxplots depicting the results from the Tukey post hoc analysis of balance beam metrics for a) time to traverse the beam, b) average box step over time, c) standard deviation of step time, and d) ratio of root-mean squared (RMS) medial-lateral (M-L) acceleration to anterior-posterior (A-P) acceleration. The bars denote significant differences between loading conditions at a significance level α = 0.05*, 0.01**, 0.001***.

Fig 10 illustrates selected results from the Tukey post hoc analyses. The time to traverse the beam increases with load and exhibits significant differences in the UL-15BW (p<0.01, *d* = -0.51) and UL-30BW (p<0.001, *d* = -0.92) comparisons, but not between the loaded conditions (Fig 10A). The average box step over time (Fig 10B) also increases with load, but only the UL-30BW comparison is significantly different (p<0.001, *d* = -0.66). The standard deviation of step time (Fig 10C) also increases with load, but again, only the UL-30BW comparison is significantly different (p = 0.04, *d* = -0.55). The ratio of RMS M-L sacrum acceleration to that of the A-P acceleration decreases with load, with significant differences in the UL-15BW (p<0.001, *d* = 1.43) and UL-30BW (p<0.01, *d* = 1.13) comparison, but not between the loaded conditions (Fig 10D).

Consistent with previous findings [33], load has significant effects on balance performance. Participants cross the beam more slowly and navigate the boxes more slowly using more varied gait to maintain balance on the beam. However, unlike the previous findings, the percentage of time spent in double support is not affected significantly by load. In the previous study [33], the obstacle had no boxes for the participants to step over. Therefore, the different finding in this study is likely due to the presence of boxes on the beam; the boxes limit the stride lengths (and therefore speed) that participants can use when traversing the beam. Finally, the addition of load suppresses variations in trunk lean in the ML direction relative to the AP direction.

### Wall

The wall obstacle is a freestanding wooden wall (1.22 m in height). Participants are instructed to quickly approach the wall and use both hands and feet to pass over the wall as quickly as possible while maintaining control of the mock rifle with at least one hand at all times. Participants begin the obstacle in a quiet standing posture (4.5 m) in front of the wall. After passing over the wall, participants run and stop (approximately 4.5 m past the wall) returning to quiet standing posture.

Performance in the wall obstacle is quantified using metrics identical to the window obstacle metrics described above: namely, 1) time to pass over the wall, 2) horizontal approach velocity, 3) vertical takeoff velocity, 4) vertical takeoff power, and 5) vertical landing velocity. The results of the ANOVA and Tukey post hoc analyses for the wall obstacle are reported in Table F in S1 File. Salient results are presented and discussed below.

The ANOVA reveals that load has significant relationships related horizontal approach velocity (F(1.4,25.7) = 9.5, p = 0.002, *η*^2^ = 0.34), vertical takeoff velocity (F(2,36) = 16.2, p<0.001, *η*^2^ = 0.47), vertical takeoff power (F(2,36) = 4.9, p = 0.013, *η*^2^ = 0.22), and vertical landing velocity does (F(2,36) = 6.6, p = 0.004, *η*^2^ = 0.27). The time to pass over the wall did not have a significant relationship with load.

**Fig 11:**
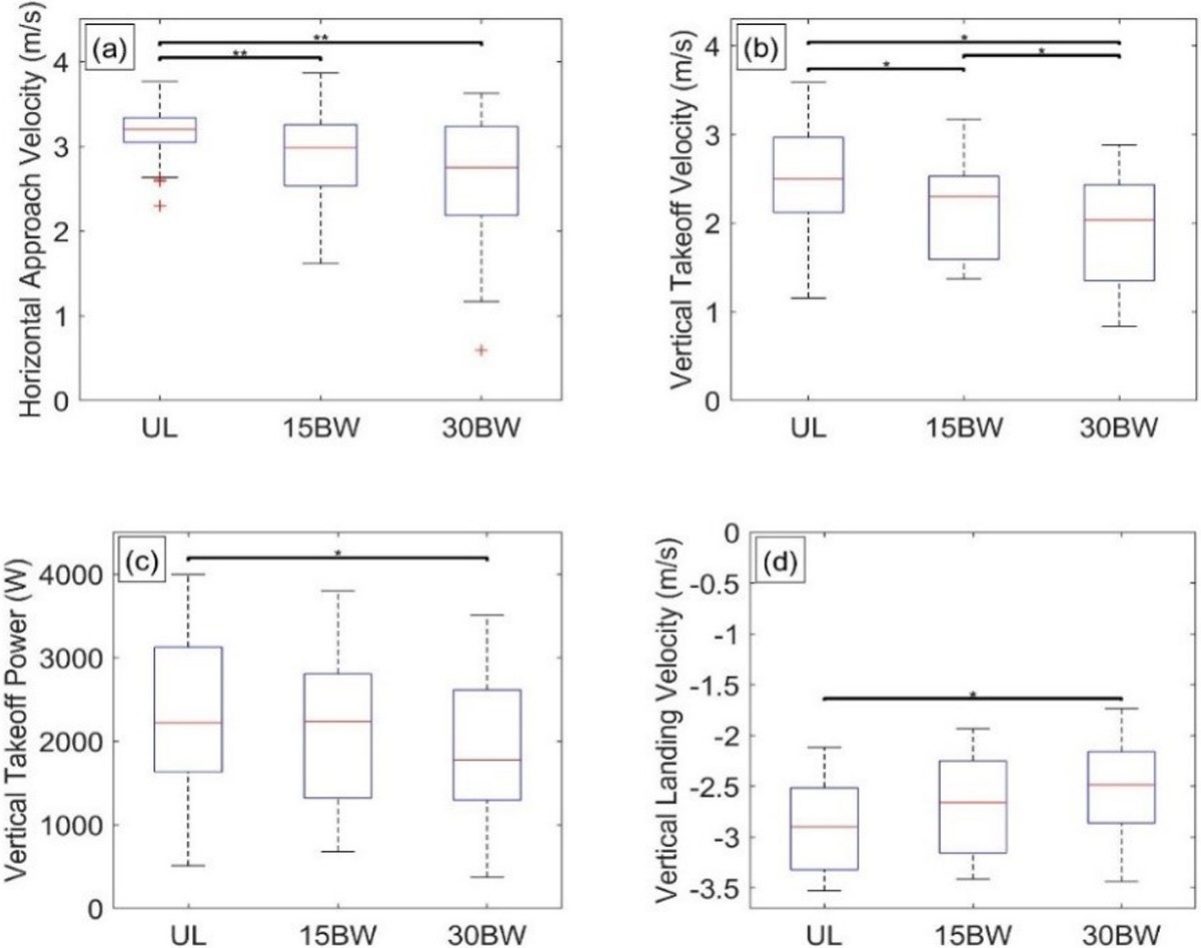
Wall statistical results. Boxplots depicting the results from the Tukey post hoc analysis of wall obstacle metrics for a) horizontal approach velocity, b) vertical takeoff velocity, c) vertical takeoff power and d) vertical landing velocity. The bars denote significant differences between loading conditions at a significance level α = 0.05*, 0.01**, 0.001***.

Fig 11 illustrates selected results from the Tukey post hoc analyses. The horizontal approach velocity decreases with load and exhibits significant differences in the UL-15BW (p<0.01, *d* = 0.63) and UL-30BW (p<0.01, *d* = 0.88) comparisons, but not between the loaded conditions (Fig 11A). All three pairwise comparisons for the vertical takeoff velocity are significant, indicating that vertical takeoff velocity decreases with added load (Fig 11B). Only the UL-30BW comparison is significant for both vertical takeoff power (p = 0.02, *d* = 0.48; Fig 11C) and vertical landing velocity (p<0.001, *d* = -0.76; Fig 11D).

The time to pass over the wall is unaffected by load. Instead, participants choose to modify their strategies when carrying load. When carrying load, participants approach the wall more slowly. Similar to the vertical jump and the window obstacle, vertical takeoff velocity decreases with load. However, unlike the vertical jump and window obstacle, vertical takeoff power also decreases with added load, suggesting that participants choose to decrease vertical takeoff power to accommodate a different strategy when climbing over the wall with load. For example, less vertical displacement (and less vertical power) is required to roll over the wall versus climbing over the wall in an upright posture; more strategies are available to participants to traverse the wall since body configurations are not constrained by a window opening, as verified by video. Finally, participants land with smaller magnitude vertical velocities when carrying load; this is also likely due to a different strategy, as lower magnitude vertical velocities require lower drop heights. For example, instead of jumping off of the wall (large drop height), participants choose to sit and drop off of the wall or roll off of the wall (smaller drop height) when carrying additional load. Participants could be mitigating injury potential and/or loads on their joints by reducing the impact with the ground.

### Agility run

**Fig 12:**
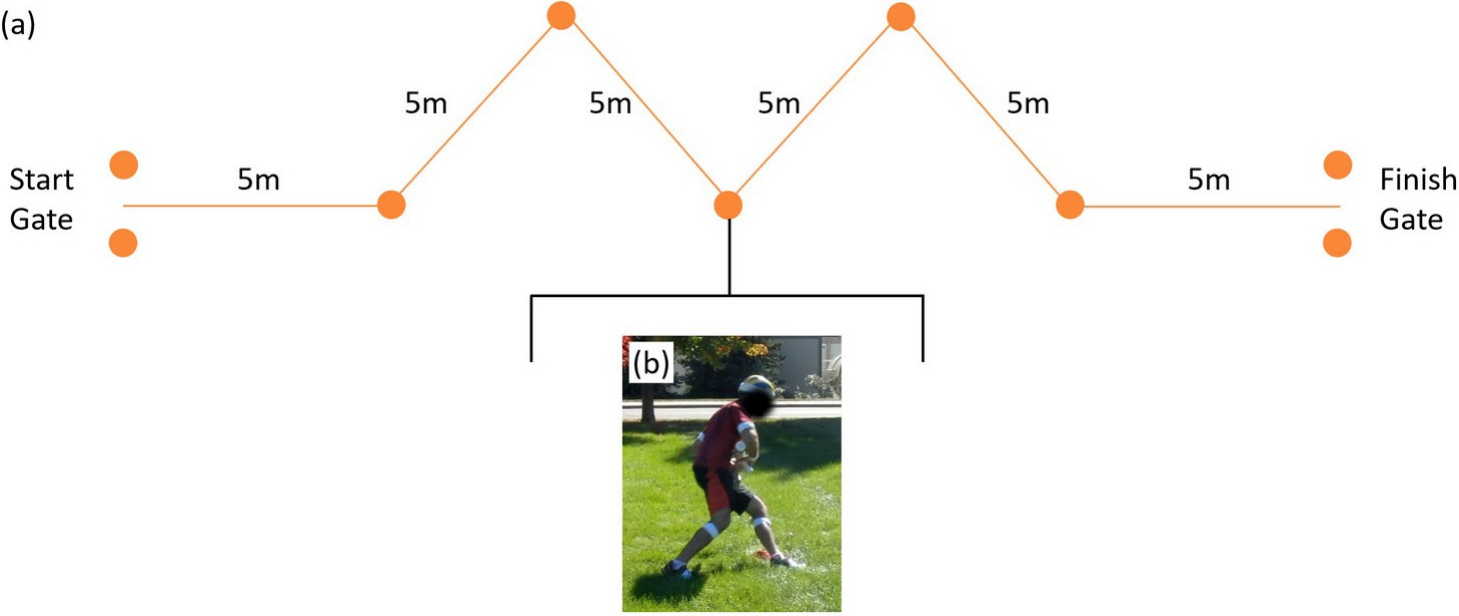
Agility run obstacle. a) The layout for the agility run obstacle and an example of a participant performing the task. The photo (b) illustrates a subject cutting around the outside of a cone.

A detailed description of the agility run and the associated performance metrics used in this study can be found in [35] and a brief summary is also provided here. Fig 12A shows the agility course setup that includes a starting gate, a finish gate and five intermediate cones. Participants run as fast as possible from the starting gate through the finish gate while cutting close to the outside of the five cones (Fig 12B). During this obstacle, participants maintained control of the mock rifle (ready position) with both hands at all times.

All agility run performance metrics are calculated using data from the foot-mounted IMUs. Similar to the method described above in the casualty drag obstacle, the foot IMU data yield estimates of the trajectory of each foot following methods similar to [43] as further specialized in [35]. Instantaneous foot trajectory, velocity, acceleration, and path curvature are calculated for each foot and then averaged across right and left foot to estimate the (instantaneous) values for the body (approximate mass center). The trajectory of the body was further decomposed into turn and straightaway phases distinguished by regions of high and low path curvature, respectively. Nine performance metrics of agility performance, reviewed briefly below, follow from this data; namely, 1) obstacle time and 2) distance traveled, 3) average body speed, 4) average turn radius, 5) average turn speed, 6) average maximum turn speed, 7) average straightaway speed, 8) average maximum straightaway speed, and 9) acceleration range. The obstacle time and distance traveled follow from the times and locations of peak path curvature detected at the first and last cones. The average body speed is the mean of the body speed across the overall obstacle distance/time traveled. The average turn radius follows from the path curvature averaged across the three inner turn phases. Average body speed and average maximum body speed during turns and straightaways are the averages and averages of the maxima across the inner three turns and inner four straightaways, respectively. Finally, the acceleration range is the maximum acceleration during a straightaway minus the minimum acceleration (maximum deceleration) along that straightaway and averaged across the inner (four) cone straightaways. Note that for the statistical analysis, the obstacle time is not normally distributed and was transformed to a normal distribution (via an inverse transform) prior to the ANOVA. The results of the ANOVA and Tukey post hoc analyses for the agility run are reported in Table G in S1 File. Significant results are presented and discussed below.

The ANOVA reveals relationships with load for the acceleration range (F(2,30) = 15.1, p<0.001, η^2^ = 0.50), (inverse) obstacle time (F(1.33,19.97) = 13.8, p<0.001, η^2^ = 0.48), average body speed (F(2,30) = 7.9, p<0.01, η^2^ = 0.35), average straightaway speed (F(2,30) = 9.2, p<0.001, η^2^ = 0.38), and the average maximum straightaway speed (F(2,30) = 14.1, p<0.001, η^2^ = 0.48). By contrast, no significant statistical relationships emerge for the average turn speed, average maximum turn speed, total distance traveled, or average turn radius.

**Fig 13:**
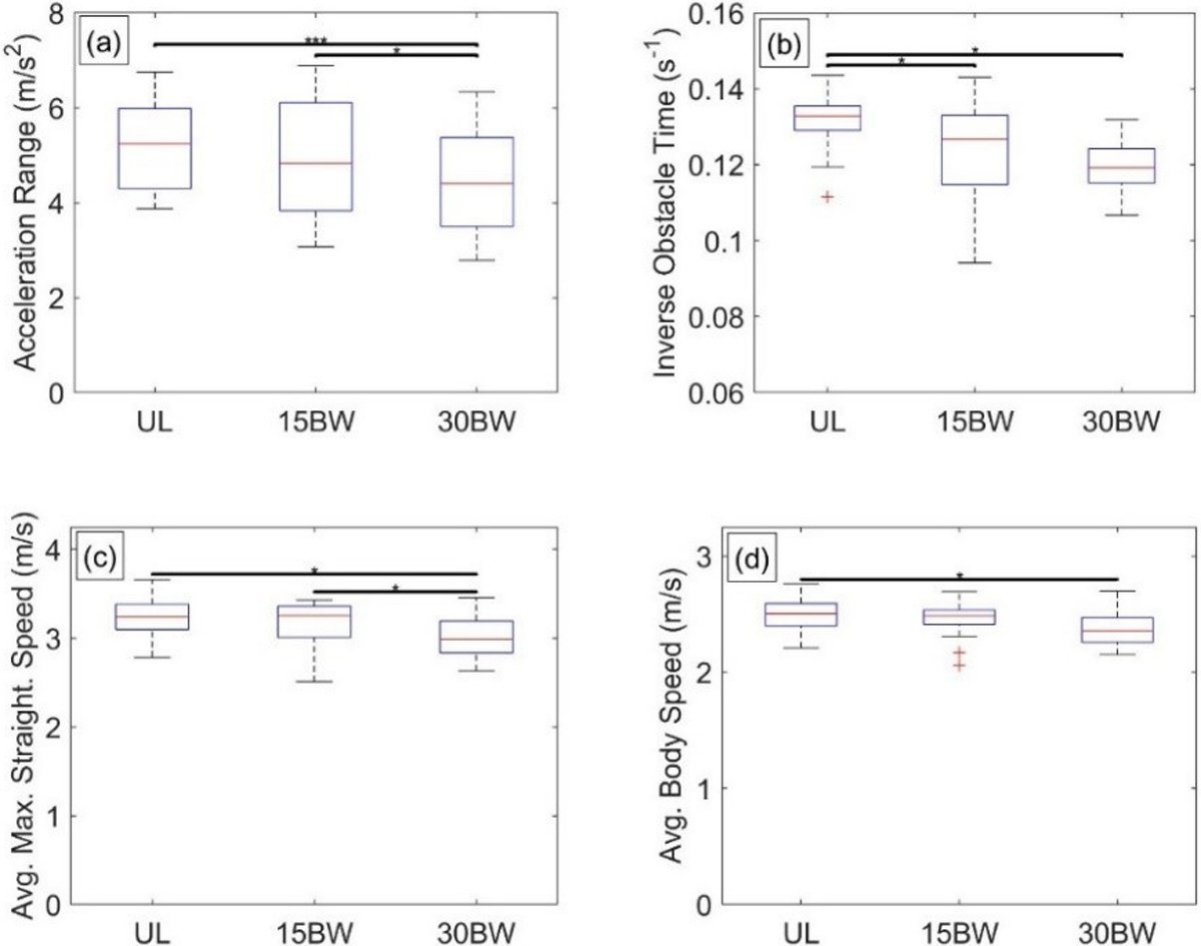
Agility run statistical results. Boxplots depicting the results from the Tukey post hoc analysis for a) acceleration range, b) (inverse) obstacle time, c) average maximum straightaway speed, and d) average body speed. The bars denote significant differences between loading conditions at a significance level α = 0.05*, 0.01**, 0.001***.

Fig 13 illustrates selected results from the Tukey post hoc analyses. In particular, Fig 13A confirms that the acceleration range decreases with load, an effect most significant in the UL-30BW comparison (p<0.001, *d* = 0.86) relative to the remaining two comparisons. This result is expected as a participant’s ability to accelerate and decelerate along a straightaway is compromised by added mass. The (inverse) obstacle time also exhibits dependence on load (Fig 13B) with significant differences arising in the UL-15BW (p<0.001, *d* = 0.71) and UL-30BW (p<0.001, *d* = 1.65) comparisons, but not between the two loaded conditions. Thus, reliance on obstacle time alone for measuring performance in the agility run may not reveal an anticipated continuous degradation in performance with increasing load.

The results for the average maximum straightaway speed (Fig 13C) closely follow the trends observed for the acceleration range, including the significant UL-30BW comparison (p<0.001, *d* = 0.95). This is not surprising given the ANOVA results indicate a strong relationship between speed and load during the straightaway phases but not during the turn phases. Participants enter and leave the turn phases with similar (low) speeds regardless of loading condition, but then accelerate to very different (high) speeds on the straightaways with load as anticipated by the strong dependence of acceleration range on load. While also significantly dependent on load, the average straightaway speed (Fig C in S1 File) follows the illustrated results for the average maximum straightaway speed (Fig 13C), including the significant UL-30BW comparison (p<0.01, *d* = 0.87). Finally, the average body speed demonstrates significant reduction with load in the UL-30BW comparison (p<0.01, *d* = 0.80), but not in the other two comparisons. The average body speed simultaneously accounts for the speed during the straightaways, which is sensitive to load, and the speed during the turns, which is largely insensitive to load. Thus, like the overall obstacle time, the overall body speed alone does not exhibit the anticipated, continuous reduction with load.

### Bounding rush

**Fig 14:**
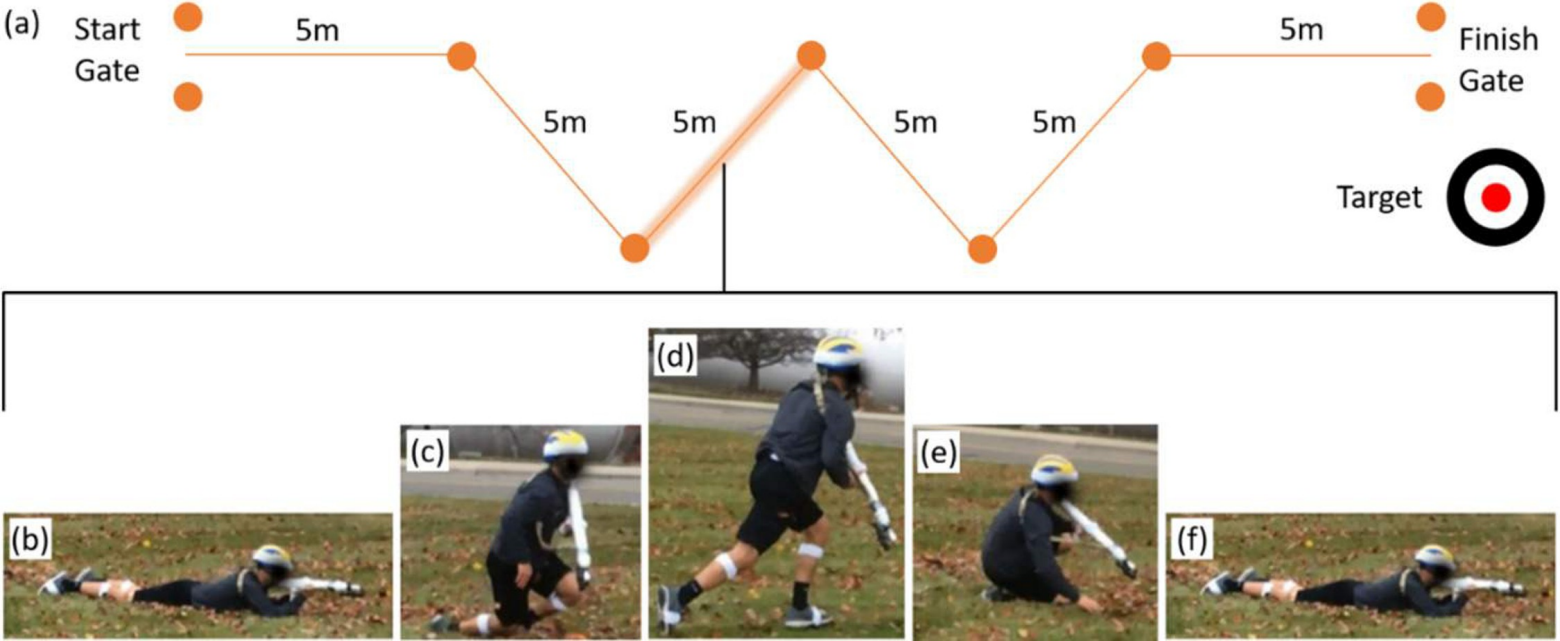
Bounding rush obstacle. a) The layout for the bounding rush obstacle and an example of a participant aiming and then executing a single bounding rush (b)-(f). The photos illustrate the aiming phases (b) and (f) that bookend each bounding rush that consists of: c) standing quickly, d) sprinting to the next cone, and e) dropping quickly to prone.

The bounding rush obstacle is performed on the agility run course wherein participants sprint from the start gate to the first cone of five cones, complete 4 bounding rushes, and then sprint to the finish gate as illustrated in Fig 14A. Between each bounding rush, participants aim the mock rifle while lying prone (participants aim at a target adjacent to the finish gate for brief participant-selected times); refer to Fig 14B and 14E. A bounding rush consists of three movement phases starting with the participant standing quickly from the prone position, sprinting to next cone, and then dropping quickly to the prone position; refer to Fig 14C–14E. Participants are instructed to maintain control of the mock rifle (ready position) with at least one hand at all times.

Performance in the bounding rush is deduced from four speed metrics and two power metrics. The performance metrics are computed from the (drift-corrected) velocity of the sacrum-mounted IMU estimated using an algorithm similar to that used to calculate the foot trajectory from a foot-mounted IMU [33, 43]. Instances when participants are prone and aiming at the target are considered still periods between which the vertical velocity is corrected for the (approximately linear) drift. From these estimates, four speed metrics are developed to measure the standing speed, dropping speed, sprinting speed, and overall speed of the bounding rush cycle. The power metrics estimate the (positive) power averaged during standing phases and the (negative) power averaged during dropping phase with the instantaneous power given by

$$P=\left(a_{S}+g\right)\left(m_{P}+m_{L}\right)v_{S}$$ (3)

Where *a*_*s*_ is the vertical acceleration of the sacrum, *g* is the acceleration due to gravity, *m*_*p*_ is the participant mass, *m*_*L*_ is the mass of the additional load, and *ν*_*S*_ is the vertical velocity of the sacrum. The six performance metrics are the averages across all four bounding rush cycles for each participant. The results of the ANOVA and Tukey post hoc analyses for the bounding rush are reported in Table H in S1 File. Salient results are presented and discussed below.

The ANOVA reveals relationships with load for overall rushing speed (F(1.4,23.7) = 22.2, p<0.001, *η*^2^ = 0.57), standing speed (F(2,34) = 16.5, p<0.001, *η*^2^ = 0.49), sprinting speed (F(2,34) = 15.3, p<0.001, *η*^2^ = 0.47), and dropping speed (F(2,34) = 7.56, p<0.01, *η*^2^ = 0.31). By contrast, no relationships with load arises for either the standing or dropping power. This is likely due to participants decreasing their vertical velocity (and also acceleration) during their standing and dropping phases in proportion to increasing load.

**Fig 15:**
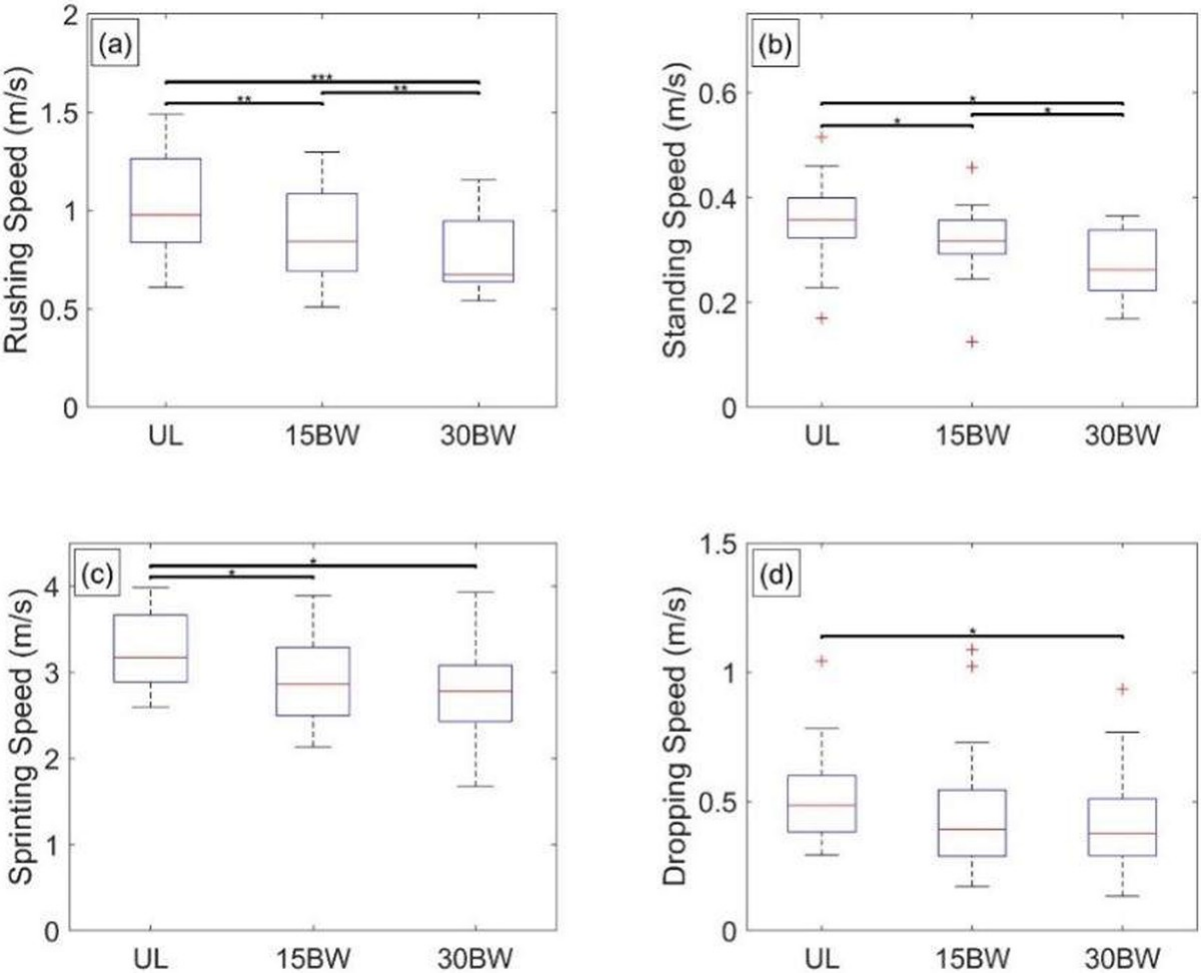
Bounding rush statistical results. Boxplots depicting the results from the Tukey post hoc analysis for a) rushing speed, b) standing speed, c) running speed, and d) dropping speed. The bars denote significant differences between loading conditions at a significance level α = 0.05*, 0.01**, 0.001***.

The significant results from the Tukey post hoc analyses are revealed in the box plots illustrated in Fig 15. In Fig 15A, all three pairwise comparisons of the overall rush speed are significant, suggesting increased difficulty of completing a bounding rush with added load. Among the speeds of the individual movement phases (refer to Fig 15B, 15C and 15D) only the standing speed (Fig 15B) exhibits significant pairwise comparisons for all three loading conditions. The standing phase requires significant upper body strength and some participants modify their standing technique due to added load. For instance, some participants with added load support themselves on one knee before fully standing, whereas without added load immediately stand (i.e. no intermediate knee support).

Sprinting speed exhibits significant differences between the UL and both loaded conditions, but not between loaded conditions (Fig 15C). Generally, the differences in the rushing speeds appear to be driven by the sprinting speed for UL-15BW comparison (p<0.01, *d* = 0.56) and by standing speed for 15BW-30BW comparison (p = 0.02, *d* = 0.66). Dropping speed (Fig 15D) exhibits only a medium-sized (p = 0.02, *d* = 0.50) significant difference for the UL-30BW comparison and is therefore not driving the overall rushing speed. However, as observed in the video recordings, many participants modify their dropping technique under the 30BW condition by first dropping to one or both knees prior to settling into the prone position. The intermediate knee support likely reduces injury potential from otherwise increased ground impact with added load.

### High crawl

**Fig 16:**
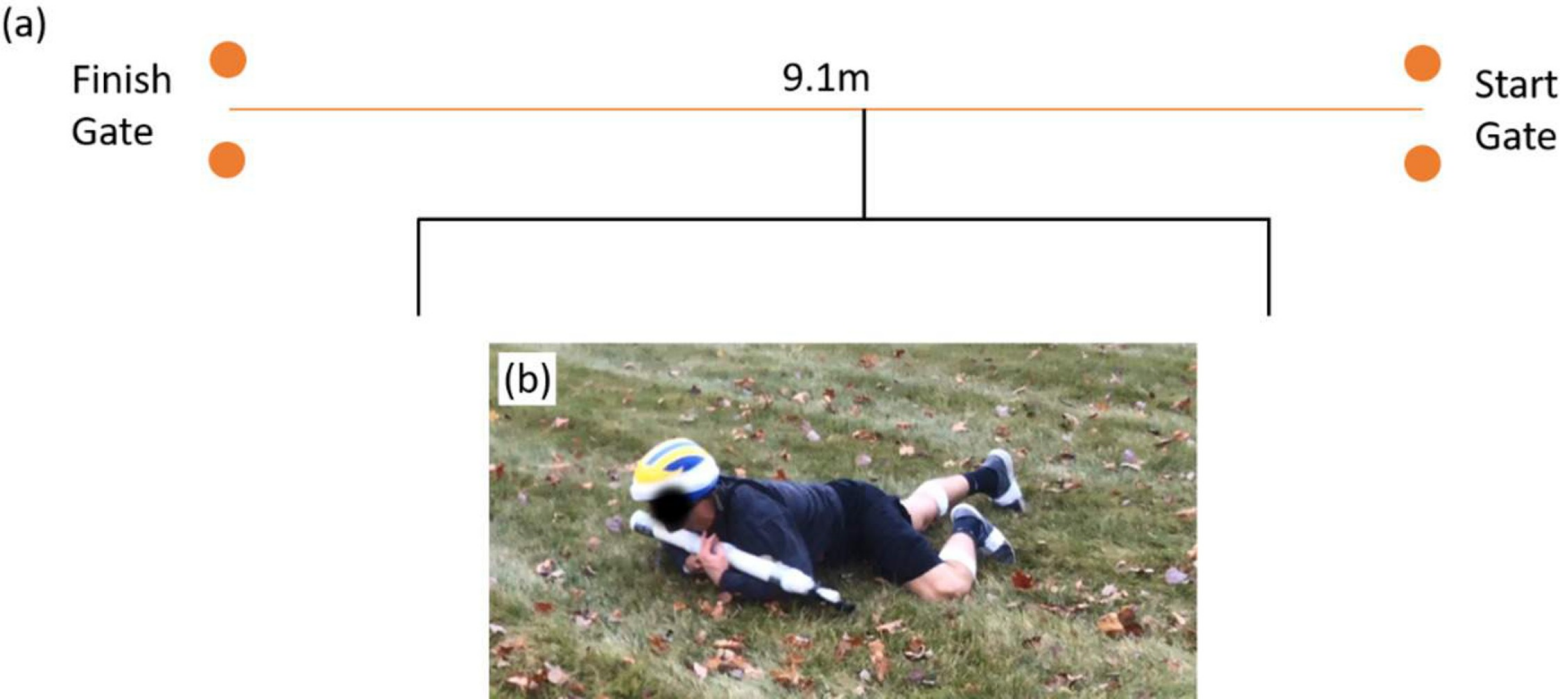
High crawl obstacle. (a) Layout of the high crawl obstacle, and (b) photograph of a participant completing the high crawl.

A detailed description of this obstacle and related performance metrics derived from upper arm-mounted and thigh-mounted IMUs is documented in [39], and a summary is offered here in the context of this load study. Participants start from rest at one cone and crawl as quickly as possible on elbows/forearms and knees to a second cone (9.1 m from first cone per Fig 16A) while supporting the mock rifle between their forearms and biceps (Fig 16B). The metrics described below are derived from the IMUs attached to the upper arms and thighs.

Performance in the high crawl is deduced from an estimated crawl speed, crawl stride time, and two crawl coordination metrics. The estimated crawl speed follows from the known length of the crawl (9.1m) divided by the crawl completion time. Large amplitude arm accelerations arise each time an elbow strikes the ground, and the associated acceleration spikes readily identify the start and end of each crawl stride (and thus also the crawl completion time). Crawl stride time is the mean of the stride times defined by those elbow strikes (averaged across the right and left elbow strikes). Two coordination metrics describe the phasing of the ipsilateral and contralateral pairs of upper arms and thighs as determined from the angular velocities of the upper arms and thighs as follows.

For each stride, the first component of a principal component analysis [44] of the upper arm or thigh angular velocities defines the principal axis of rotation for that body segment. The angular velocity for each upper arm projected onto the associated principal axis of rotation yields the principal angular speed *ω*_*arm*,*i*_ where *i = l* or *r* for *left* or *right* upper arm. A similar procedure is followed for each thigh but conducted twice, once using strides defined by the left elbow strikes and once using strides defined by the right elbow strikes. Doing so yields the principal angular speed *ω*_*leg*,*ij*_ where *i = l* or *r* for *left* or *right* elbow strikes and *j = l* or *r* for *left* or *right* thigh. Next, the phasing of each pair of arm and leg principal angular speeds is defined as

$$\beta_{ij}=\frac{\int_{t_{1}}^{t_{2}}\omega_{arm,i}\left(t\right)\omega_{leg,ij}\left(t\right)dt}{\sqrt{\int_{t_{1}}^{t_{2}}\omega_{arm,i}{\left(t\right)}^{2}dt}\sqrt{\int_{t_{1}}^{t_{2}}\omega_{leg,ij}{\left(t\right)}^{2}dt}}$$ (4)

where the limits of integration begin with the time of the first elbow strike (t_1_) to the last elbow strike (t_2_), i.e., the crawl completion time. The component *β*_*ij*_ is a measure of the phase between the principal angular speeds of the *i*^*th*^ upper arm and the *j*^*th*^ thigh (calculated with the *i*^*th*^ elbow strikes), where again *i*, *j = l(eft)* or *r(ight)*. Note that −1 ≤ *β*_*ij*_ ≤ 1 and that the limiting values *β*_*ij =*_ 1 and *β*_*ij*_ *=* −1 denote perfectly in-phase and perfectly out-of-phase motions, respectively. Leveraging this observation, we define two coordination metrics; namely, the ipsilateral limb coordination as

$$\beta_{i}=\frac{\beta_{armr,legr}+\beta_{arml,legl}}{2}$$ (5)

and contralateral limb coordination as

$$\beta_{c}=\frac{\beta_{armr,legl}+\beta_{arml,legr}}{2}$$ (6)

The results of the ANOVA and Tukey post hoc analyses for the high crawl are reported in Table I in S1 File. Salient results are presented and discussed below.

The ANOVA reveals significant relationships with load for all four crawl performance metrics: crawl speed (F(1.4,18.5) = 43.2, p<0.001, *η*^2^ = 0.77), crawl stride time (F(2,26) = 18.1, p<0.001, *η*^2^ = 0.58), contralateral coordination (F(2,26) = 9.44, p<0.001, *η*^2^ = 0.42), and ipsilateral coordination (F(2,26) = 4.25, p = 0.03, *η*^2^ = 0.25).

**Fig 17:**
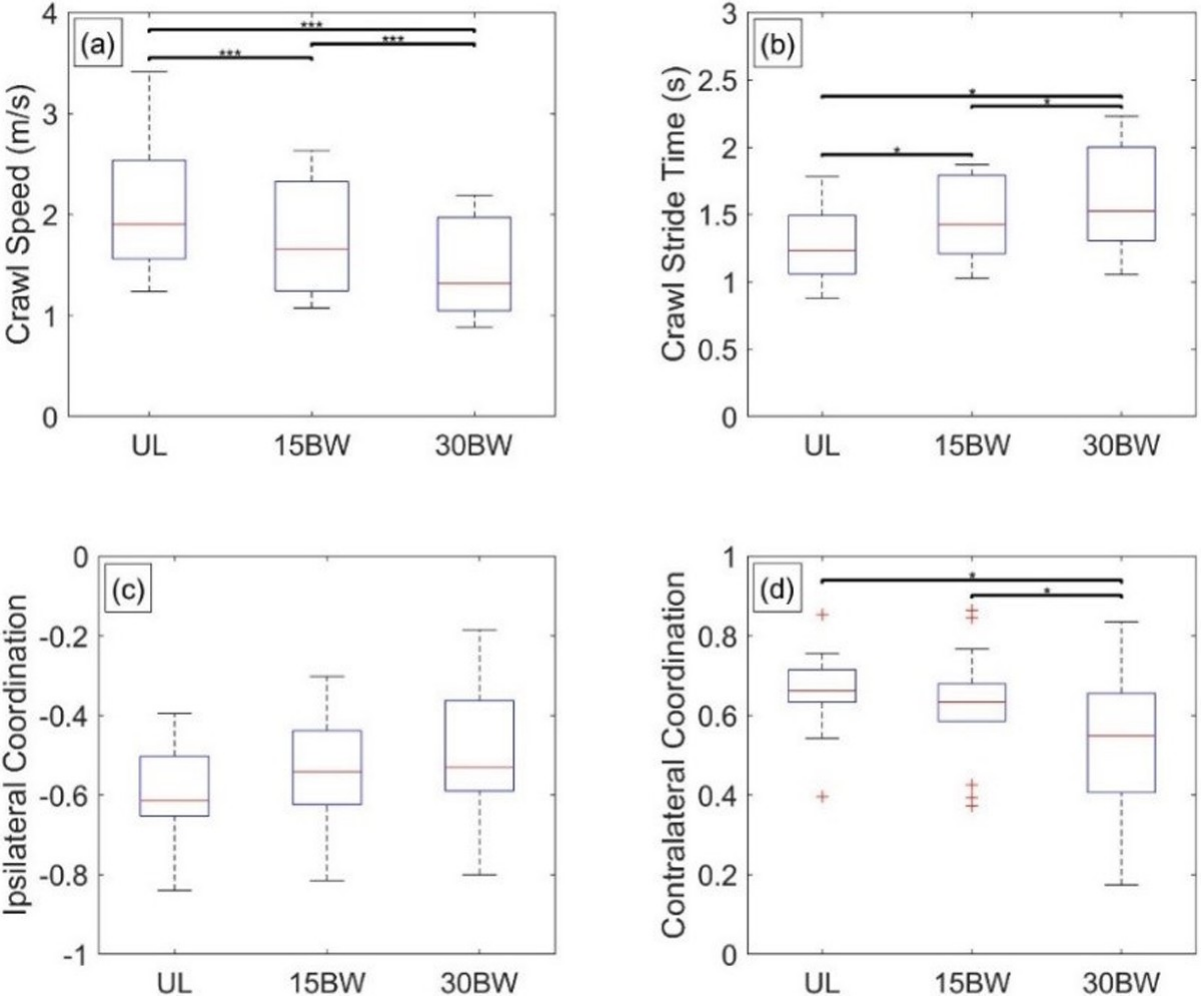
High crawl statistical results. Boxplots depicting the results from the Tukey post hoc analysis for a) crawl speed, b) crawl stride time, c) ipsilateral coordination, and d) contralateral coordination. The bars denote significant differences between loading conditions at a significance level α = 0.05*, 0.01**, 0.001***.

Results from the Tukey post hoc analyses are illustrated in the box plots in Fig 17. The three pairwise comparisons for the crawl speed are significant, implying the added loads increase the difficulty of the crawling task manifesting in significantly slower crawl speeds (Fig 17A). The three pairwise comparisons for the crawl stride time are also significant (Fig 17B), but the effect sizes could indicate a potentially nonlinear increase in stride time with load. In particular, the effect size for the UL-15BW comparison (p<0.01, *d* = -0.68) is nearly twice that of the 15BW-30BW comparison (p = 0.03, *d* = -0.39).

In this context, superior crawling performance is characterized by a diagonal stride pattern where elbows and knees on opposite sides of the body are in simultaneous ground contact [39]. This strategy results in ipsilateral limbs moving largely out-of-phase and contralateral limbs moving largely in-phase to produce faster crawl speeds and shorter crawl stride times [39]. Even though the ANOVA results for the ipsilateral coordination indicate a relationship with load, the Tukey analysis did not reveal any significant differences (Fig 17C). This is likely due to variation in technique meaning changes in this coordination metric may not be consistent across participants. The medium-large effect size for the UL-30BW comparison (p = 0.07, *d* = -0.67) for ipsilateral coordination suggests load overall tends to reduce the degree to which the ipsilateral limbs move out-of-phase. Contralateral coordination, however, has significant relationships for the UL-30BW comparison as well as the 15BW-30BW comparison, which implies a possible nonlinear effect between load and the coordination of the contralateral limbs (Fig 17D). In particular, the effect size for the 15BW-30BW comparison (p = 0.04, *d* = 0.49) is nearly twice that of the UL-15BW comparison (p = 0.27, *d* = 0.28). This could mean the 30BW condition is large enough that participants are unwilling and/or unable to support the added weight with two points of contact and instead maximize the time they have with three points of contact. This change in strategy is marked by a simultaneously decrease in contralateral coordination and increase in ipsilateral coordination yielding longer periods of three-point contact [45].

### Vertical transfer

A detailed description of the vertical transfer obstacle and related performance metrics derived from a load-embedded IMU is documented in [37], and a summary is offered here in the context of this load study. The vertical transfer obstacle requires participants to lift the load (mass 13.6 kg) from the ground to the top of a stand (height 1.67 m). Participants are instructed to lift the load as rapidly as possible while momentarily releasing their hand(s) from the load when it first contacts the top of the stand and the ground. Participants repeat this lift/lower cycle five times with the load positioned to their right side (for a right-to-left lift) and five times with the load positioned to their left side (for a left-to-right lift). During this obstacle, the mock rifle is slung across the back.

Performance in the vertical transfer obstacle is quantified by metrics obtained from an IMU embedded within the load (i.e. within the loaded ammo can). The IMU data yields estimates of the vertical velocity of the load during each lift and lowering cycle from which we define four performance metrics: duration of lift, duration of lowering, lift smoothness, and lowering smoothness [37]. The durations of the lift and lowering follow from the vertical velocity as follows. The start time of the movement (lift or lowering) is detected when the vertical velocity first exceeds zero and the end time of the movement is detected when the vertical velocity returns to zero. The lift/lowering smoothness measures the normalized root-mean-square difference between the measured vertical velocity profile and an optimal velocity profile that minimizes jerk (see [37] for details).

The performance metrics from all ten lift/lowering cycles are averaged to yield mean values recognizing that no differences were previously observed for right-to-left lifts versus left-to-right lifts [37]. The ANOVA (results reported in Table J in S1 File) reveals that load does not have a significant relationship with any of the performance metrics, indicating that added load (carried by a weight vest) has no significant effect on vertical transfer performance. This is likely because the load mass (the ammo can) dominates this lifting task, and accelerations and movements of the thorax during the task remain small relative to the accelerations and movements of the upper limbs (and ammo can).

## Summary and conclusions

Military organizations worldwide employ standardized obstacle courses that embed combat relevant movements and tasks to understand the effects of added load on warfighter performance. To date, performance has been quantified solely by the recorded times to complete the course and any obstacle within. This study contributes a new method to quantify performance and, importantly, a new way to discriminate performance under added load. In the new method, wearable IMUs measure the underlying movements of the major body segments that define and limit performance.

**Fig 18:**
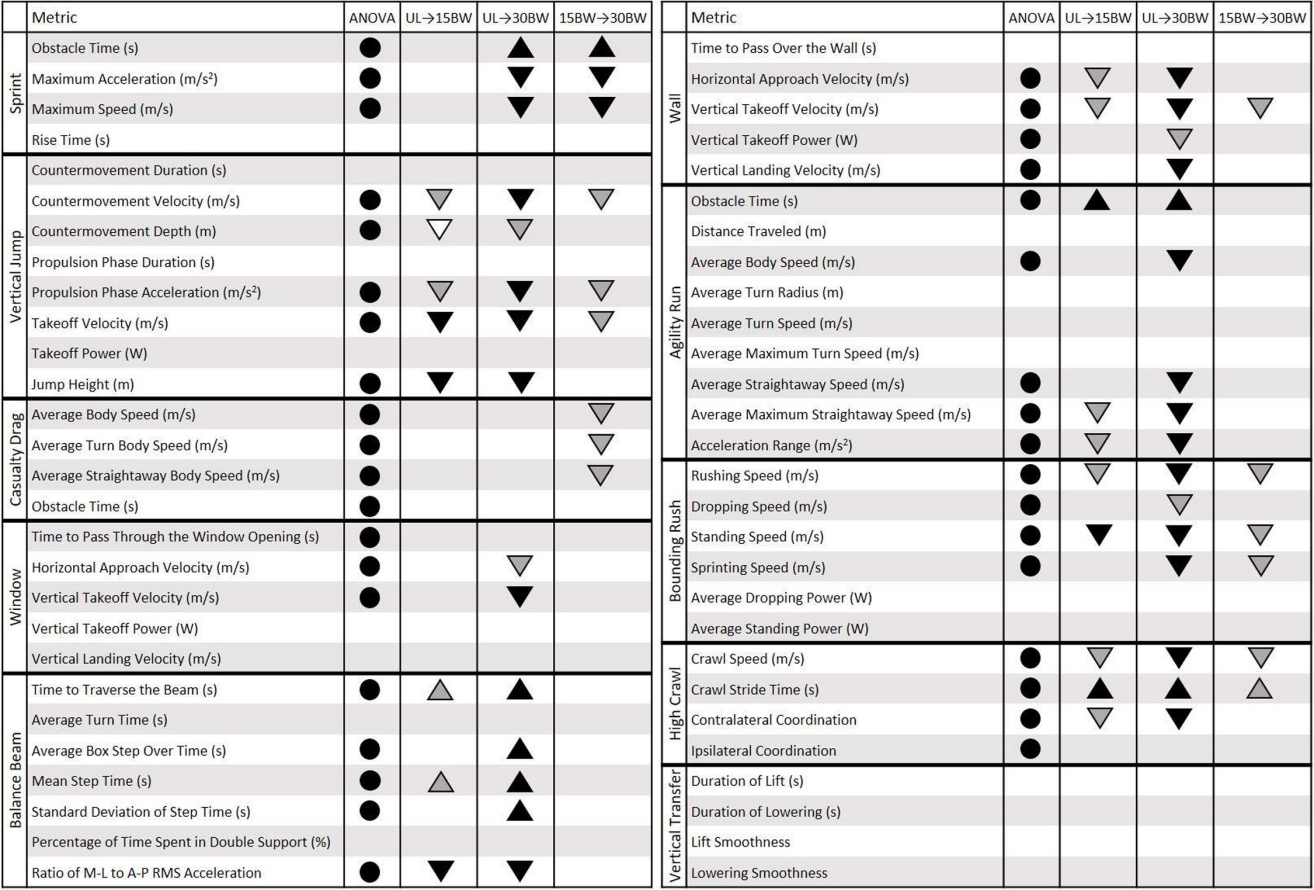
Summary ANOVA and Tukey post hoc results for all performance metrics across all obstacles. The circles under ANOVA indicate metrics exhibiting statistically significant relationships with load. Triangles under the three load comparisons (UL →15BW, UL →30BW, and 15BW →30BW) indicate statistically significant differences between load pairs (Tukey post hoc analysis). The direction of the triangle (up or down) indicates the direction of the change (increase or decrease) in the performance metric (from the first listed loading condition to the second listed loading condition). The gray scale for both symbols (circles and triangles) indicate the effect size defined in Table 2 (white = small effect size, gray = medium effect size, black = large effect size).

The new method is demonstrated on a study of (N = 22) participants who wore an array of 13 IMUs attached to major body segments (head, sternum, upper arms, forearms, sacrum, thighs, shanks, and feet) while completing an obstacle course. The obstacle course included ten obstacles referred to as the 1) sprint, 2) vertical jump, 3) casualty drag, 4) wall, 5) balance beam, 6) window, 7) agility run, 8) bounding rush, 9) high crawl, and 10) vertical transfer. Participants completed the obstacle course under four conditions (twice without added load and twice with loads of 15% and 30% of their body weight, yielding a total of 88 trials). IMU-derived performance metrics, specialized to each obstacle, were examined for the effects of load by an ANOVA and with a post-hoc Tukey pairwise-comparison for metrics exhibiting significant relationships with load. Fig 18 below summarizes the statistical results for all performance metrics across all obstacles.

Overall, we hypothesize and confirm that the obstacle-specific metrics of performance are affected by load. As observable in Fig 18, the ANOVA reveals significant relationships with load for the great majority of performance metrics for nine of the ten obstacles; refer to black circles that indicate large effect size. Observe further the marked degradations in performance with added load for eight of the ten obstacles; refer to black triangles that indicate large effect sizes for the Tukey post hoc comparison across load pairs and that triangle directions point toward performance degradation. Among these eight obstacles, the performance degradations present as follows. In the sprint, added load significantly increases sprint time by decreasing both the maximum acceleration and the maximum speed. In the vertical jump, added load significantly affects the countermovement phase (reducing both countermovement depth and velocity) as well as the propulsion phase (reducing the acceleration and the resulting takeoff velocity) leading to lower jump height. In both the window and wall, added load reduces the speed of approach (to the window and wall) and the vertical take-off speed (onto the window sill and the top of the wall). In addition, for the wall, added load reduces the vertical landing velocity. In the balance beam, added load increases the time to traverse the beam by increasing mean step time and the time to step over obstacles (boxes), induces greater variations in gait (step time), and decreases M-L torso acceleration over A-P torso acceleration. In the agility run, added load increases agility time by decreasing average body speed. Along the straightaways, this manifests as decreased average straightaway speed, maximum straightaway speed, and acceleration range. In the bounding rush, added load decreasing the rushing speed and particularly the speeds of the standing and sprinting phases. Finally, in the high crawl, added load decreases contralateral limb coordination thereby increasing crawl stride time and decreasing crawl speed. The remaining two obstacles did not exhibit the same trends of marked degradations in IMU-derived performance metrics with increasing load. For the casualty drag, the differences observed in the statistical analyses are non-monotonic in that added load is associated with improved as well as degraded performance depending on the load condition. For the vertical transfer, performance is not significantly related to added load.

In conclusion, data harvested from wearable IMU arrays reveal the underlying biomechanical movements that define and limit performance across multiple tasks in an outdoor obstacle course. The data yield obstacle-specific metrics of performance for quantifying the effects of load on performance. The resulting performance metrics cannot be deduced using traditional timing gate data alone. From the known metric development for the obstacles described previously, sensor placement for other obstacles studied in the future may be analytically formulated based on the research question of interest. Furthermore, while this paper considers the relationship between load and obstacle-specific performance metrics, it does not explicitly consider different strategies adopted by participants in response to the added load. However, the metrics presented herein could also be used to study additional hypotheses, as was done for the agility run [34] and high crawl [39].

## Supporting information

S1 File Statistical results and additional information. Complete statistical results for all performance metrics for all obstacles and additional information for specific obstacles are included in this document.
(DOCX)

S2 File Results data file. Data for each performance metric from all obstacles are included in this file. It should be noted that subject codes are randomized between obstacles (e.g., Subject 1 is not necessarily the same for all obstacles).
(XLSX)
