# Supplementary material for: Body-worn IMU array reveals effects of load on performance in an outdoor obstacle course
Source: PLoS One. 2019 Mar 21;14(3):e0214008. doi: 10.1371/journal.pone.0214008 (PMC6428270; doi:10.1371/journal.pone.0214008)
Supplement: S1 File — Complete statistical results for all performance metrics for all obstacles and additional information for specific obstacles are included in this document. (DOCX) [file pone.0214008.s001.docx]

**S1 Appendix**

The tables below include the results from the repeated measures ANOVA (F-statistics with degrees of freedom, p-values, and effect sizes) and results from the Tukey post hoc analyses (p-values and effect sizes) for the performance metrics with significant ANOVA results. It should be noted that degrees of freedom that are not whole numbers are the result of a Greenhouse-Geiser correction to account for a violation of assumed sphericity.

*Sprint*

**Table A.** **Sprint statistical results.** Results from the repeated-measures ANOVA (F-statistics with degrees of freedom, p-values, and effect sizes, $\eta^{2}$). For performance metrics with significant ANOVAs, Tukey post hoc analyses are also reported (p-values and effect sizes, *d*).

|  | **ANOVA** | | | **Tukey Post Hoc** | | | | | |
| --- | --- | --- | --- | --- | --- | --- | --- | --- | --- |
|  | **F-Statistic** | **p-value** | $\boldsymbol{\eta}^{\boldsymbol{2}}$ | **0%-15%** | | **0%-30%** | | **15%-30%** | |
|  |  |  |  | **p-value** | ***d*** | **p-value** | ***d*** | **p-value** | ***d*** |
| *Obstacle Time (s)* | F(2,20)  = 17.2 | <0.001*^§^* | 0.63 | 0.07 | -0.62 | <0.01*^†^* | -1.54 | <0.01*^†^* | -1.00 |
| *Maximum Acceleration (m/s^2^)* | F(2,20)  = 14.9 | <0.001*^§^* | 0.60 | 0.16 | 0.40 | <0.01*^†^* | 1.31 | <0.01*^†^* | 0.90 |
| *Maximum Speed (m/s)* | F(2,20)  = 14.4 | <0.001*^§^* | 0.59 | 0.17 | 0.51 | <0.001*^§^* | 1.62 | 0.03* | 0.83 |
| *Rise Time (s)* | F(1.1,10.9)  = 0.76 | 0.41 | 0.07 | - | - | - | - | - | - |

*Significant at α = 0.05*, 0.01^†^, 0.001^§^*

*
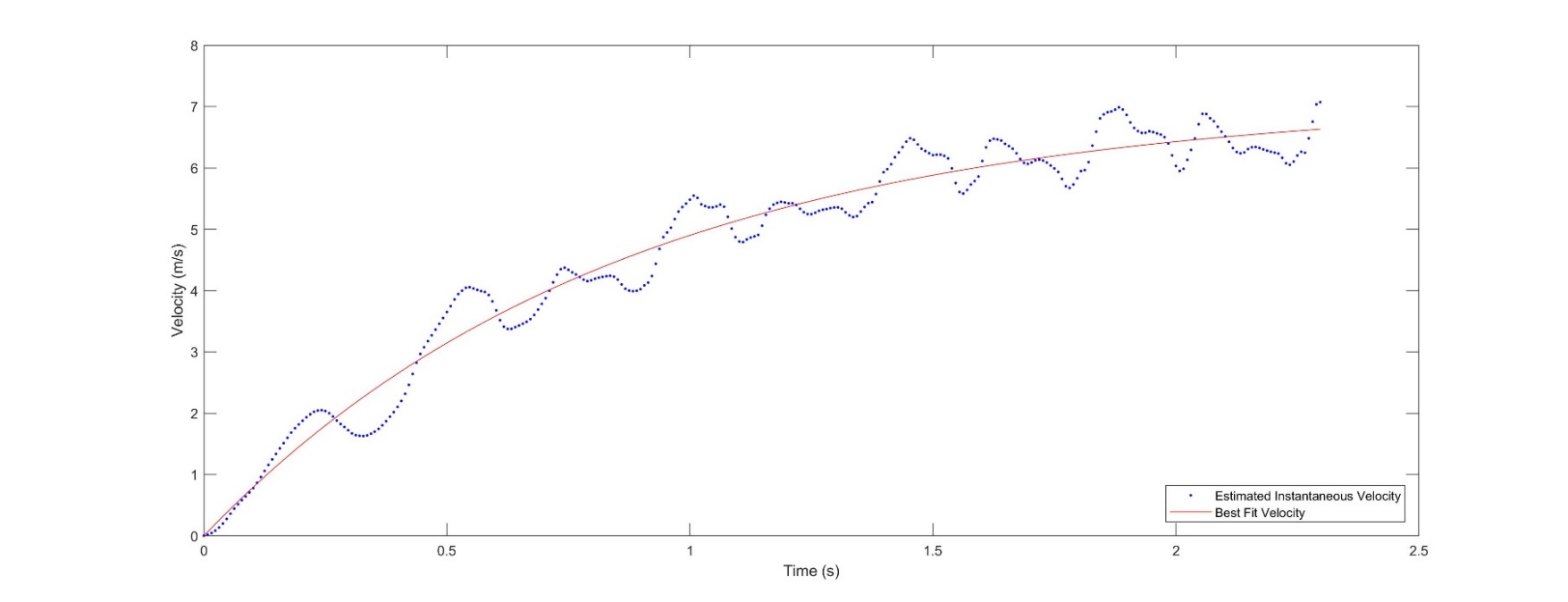
*

**Fig A.** **Horizontal body with a fitted exponential curve.** The RMSE value for the exponential fit is 0.351 m/s.

*Vertical Jumps*

**Table B. Vertical jump statistical results.** Results from the repeated-measures ANOVA (F-statistics with degrees of freedom, p-values, and effect sizes, $\eta^{2}$). For performance metrics with significant ANOVAs, Tukey post hoc analyses are also reported (p-values and effect sizes, *d*).

|  | **ANOVA** | | | **Tukey Post Hoc** | | | | | |
| --- | --- | --- | --- | --- | --- | --- | --- | --- | --- |
|  | **F-Statistic** | **p-value** | $\boldsymbol{\eta}^{\boldsymbol{2}}$ | **0%-15%** | | **0%-30%** | | **15%-30%** | |
|  |  |  |  | **p-value** | ***d*** | **p-value** | ***d*** | **p-value** | ***d*** |
| *Counter-movement Duration (s)* | F(2,36)  = 0.9 | 0.40 | 0.05 | - | - | - | - | - | - |
| *Counter-movement Velocity (m/s)* | F(1.5,27.7)  = 20.4 | <0.001*^§^* | 0.53 | 0.001*^§^* | 0.49 | <0.001*^§^* | 1.01 | 0.01*** | 0.54 |
| *Counter-movement Depth (m)* | F(2,36)  = 8.2 | 0.001*^§^* | 0.31 | 0.01*** | 0.34 | <0.01*^†^* | 0.59 | 0.30 | 0.26 |
| *Propulsion Phase Duration (s)* | F(1.2,21.1) = 1.31 | 0.27 | 0.07 | - | - | - | - | - | - |
| *Propulsion Phase Acceleration (m/s^2^)* | F(1.3,24.3) = 33.1 | <0.001*^§^* | 0.65 | <0.001*^§^* | 0.53 | <0.001*^§^* | 1.03 | 0.01*** | 0.47 |
| *Takeoff Velocity (m/s)* | F(1.3,22.8) = 63.0 | <0.001*^§^* | 0.78 | <0.001*^§^* | 0.78 | <0.001*^§^* | 1.17 | 0.001*^§^* | 0.43 |
| *Takeoff Power (W)* | F(2,36) = 1.1 | 0.35 | 0.06 | - | - | - | - | - | - |
| *Jump Height (m)* | F(1.6,28.6) = 17.0 | <0.001*^§^* | 0.49 | <0.01*^†^* | 0.80 | <0.01*^†^* | 0.93 | 0.49 | 0.23 |

*Significant at α = 0.05*, 0.01^†^, 0.001^§^*

*
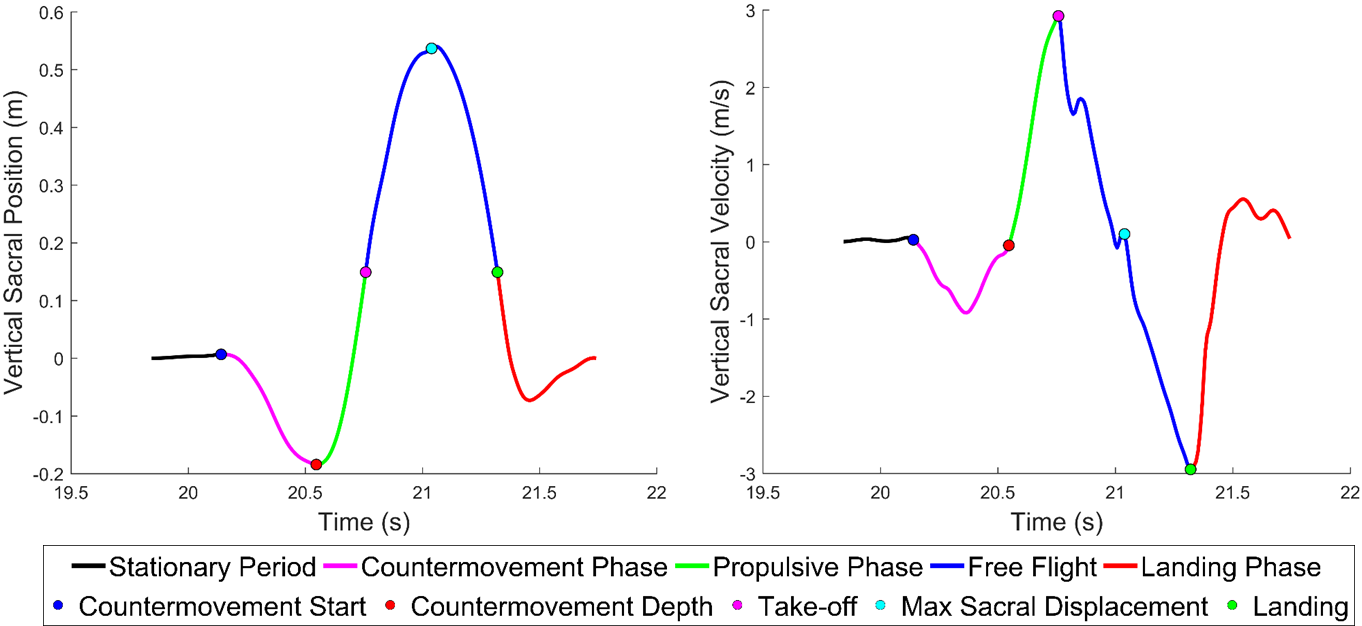
*

**Fig B. Vertical sacral position and velocity.** Each of the phases of a countermovement vertical jump is identified in both plots.

*Casualty Drag*

**Table C. Casualty Drag statistical results.** Results from the repeated-measures ANOVA (F-statistics with degrees of freedom, p-values, and effect sizes, $\eta^{2}$). For performance metrics with significant ANOVAs, Tukey post hoc analyses are also reported (p-values and effect sizes, *d*).

|  | **ANOVA** | | | **Tukey Post Hoc** | | | | | |
| --- | --- | --- | --- | --- | --- | --- | --- | --- | --- |
|  | **F-Statistic** | **p-value** | $\boldsymbol{\eta}^{\boldsymbol{2}}$ | **0%-15%** | | **0%-30%** | | **15%-30%** | |
|  |  |  |  | **p-value** | ***d*** | **p-value** | ***d*** | **p-value** | ***d*** |
| *Avg. Body Speed (m/s)* | F(2,24)  = 7.8 | <0.01*^†^* | 0.39 | 0.09 | -0.21 | 0.13 | 0.19 | 0.02* | 0.39 |
| *Avg. Turn Body Speed (m/s)* | F(2,24)  = 5.8 | <0.01*^†^* | 0.33 | 0.09 | -0.22 | 0.24 | 0.21 | 0.04* | 0.43 |
| *Avg. Straight. Body Speed (m/s)* | F(2,24)  = 5.7 | <0.01*^†^* | 0.32 | 0.19 | -0.19 | 0.17 | 0.21 | 0.04* | 0.40 |
| *Obstacle Time (s)* | F(1.2,13.9)  = 4.9 | 0.04* | 0.29 | 0.15 | 0.16 | 0.13 | -0.20 | 0.09 | -0.36 |

*Significant at α = 0.05*, 0.01^†^, 0.001^§^*

*Window*

**Table D. Window obstacle statistical results.** Results from the repeated-measures ANOVA (F-statistics with degrees of freedom, p-values, and effect sizes, $\eta^{2}$). For performance metrics with significant ANOVAs, Tukey post hoc analyses are also reported (p-values and effect sizes, *d*).

|  | **ANOVA** | | | **Tukey Post Hoc** | | | | | |
| --- | --- | --- | --- | --- | --- | --- | --- | --- | --- |
|  | **F-Statistic** | **p-value** | $\boldsymbol{\eta}^{\boldsymbol{2}}$ | **0%-15%** | | **0%-30%** | | **15%-30%** | |
|  |  |  |  | **p-value** | ***d*** | **p-value** | ***d*** | **p-value** | ***d*** |
| *Time to Pass Through the Window Opening (s)* | F(1.0,18.7)  = 6.4 | 0.02*** | 0.26 | 0.06 | -0.68 | 0.05 | -0.75 | 0.06 | -0.28 |
| *Horizontal Approach Velocity (m/s)* | F(2,36) = 5.6 | <0.01*^†^* | 0.24 | 0.07 | 0.51 | <0.01*^†^* | 0.59 | 0.75 | 0.12 |
| *Vertical Takeoff Velocity (m/s)* | F(1.5,27.5) = 7.3 | <0.01*^†^* | 0.29 | 0.09 | 0.48 | <0.01*^†^* | 0.71 | 0.55 | 0.15 |
| *Vertical Takeoff Power (W)* | F(3,54) = 2.1 | 0.11 | 0.10 | - | - | - | - | - | - |
| *Vertical Landing Velocity (m/s)* | F(2,36) = 2.0 | 0.15 | 0.10 | - | - | - | - | - | - |

*Significant at α = 0.05*, 0.01^†^, 0.001^§^*

*Balance Beam*

**Table E.** **Balance beam obstacle statistical results.** Results from the repeated-measures ANOVA (F-statistics with degrees of freedom, p-values, and effect sizes, $\eta^{2}$). For performance metrics with significant ANOVAs, Tukey post hoc analyses are also reported (p-values and effect sizes, *d*).

|  | **ANOVA** | | | **Tukey Post Hoc** | | | | | |
| --- | --- | --- | --- | --- | --- | --- | --- | --- | --- |
|  | **F-Statistic** | **p-value** | $\boldsymbol{\eta}^{\boldsymbol{2}}$ | **0%-15%** | | **0%-30%** | | **15%-30%** | |
|  |  |  |  | **p-value** | ***d*** | **p-value** | ***d*** | **p-value** | ***d*** |
| *Time to Traverse the Beam (s)* | F(2,30) = 20.6 | <0.001*^§^* | 0.58 | <0.01*^†^* | -0.51 | <0.001*^§^* | -0.92 | 0.08 | -0.31 |
| *Average Turn Time (s)* | F(1.5,22.0) = 2.8 | 0.09 | 0.16 | - | - | - | - | - | - |
| *Average Box Step Over Time (s)* | F(1.3,19.7) = 7.4 | <0.01*^†^* | 0.33 | 0.08 | -0.45 | <0.001*^§^* | -0.66 | 0.80 | -0.09 |
| *Mean Step Time (s)* | F(2,30) = 10.9 | <0.001*^§^* | 0.42 | 0.02*** | -0.37 | 0.001*^§^* | -0.57 | 0.22 | -0.23 |
| *Standard Deviation of Step Time (s)* | F(2,30) = 4.01 | 0.03*** | 0.21 | 0.12 | -0.35 | 0.04*** | -0.55 | 0.62 | -0.20 |
| *Percentage of Time Spent in Double Support (%)* | F(2,30) = 2.3 | 0.11 | 0.14 | - | - | - | - | - | - |
| *Ratio of M-L to A-P RMS Acceleration* | F(1.3,19.3) = 15.7 | <0.001*^§^* | 0.51 | <0.001*^§^* | 1.43 | <0.01*^†^* | 1.13 | 0.96 | -0.03 |

*Significant at α = 0.05*, 0.01^†^, 0.001^§^*

*Wall*

**Table F.** **Wall obstacle statistical results.** Results from the repeated-measures ANOVA (F-statistics with degrees of freedom, p-values, and effect sizes, $\eta^{2}$). For performance metrics with significant ANOVAs, Tukey post hoc analyses are also reported (p-values and effect sizes, *d*).

|  | **ANOVA** | | | **Tukey Post Hoc** | | | | | |
| --- | --- | --- | --- | --- | --- | --- | --- | --- | --- |
|  | **F-Statistic** | **p-value** | $\boldsymbol{\eta}^{\boldsymbol{2}}$ | **0%-15%** | | **0%-30%** | | **15%-30%** | |
|  |  |  |  | **p-value** | ***d*** | **p-value** | ***d*** | **p-value** | ***d*** |
| *Time to Pass Over the Wall (s)* | F(1.3,23.5) = 3.2 | 0.08 | 0.15 | - | - | - | - | - | - |
| *Horizontal Approach Velocity (m/s)* | F(1.4,25.7) = 9.5 | <0.01*^†^* | 0.34 | <0.01*^†^* | 0.63 | <0.01*^†^* | 0.88 | 0.15 | 0.38 |
| *Vertical Takeoff Velocity (m/s)* | F(2,36) = 16.2 | <0.001*^§^* | 0.47 | 0.04*** | 0.45 | <0.001*^§^* | 0.83 | 0.01*** | 0.42 |
| *Vertical Takeoff Power (W)* | F(2,36) = 4.9 | 0.01*** | 0.22 | 0.29 | 0.20 | 0.02*** | 0.48 | 0.24 | 0.28 |
| *Vertical Landing Velocity (m/s)* | F(2,36) = 6.6 | <0.01*^†^* | 0.27 | 0.34 | -0.34 | <0.001*^§^* | -0.76 | 0.17 | -0.40 |

*Significant at α = 0.05*, 0.01^†^, 0.001^§^*

*Agility Run*

**Table G. Agility run statistical results.** Results from the repeated-measures ANOVA (F-statistics with degrees of freedom, p-values, and effect sizes, $\eta^{2}$). For performance metrics with significant ANOVAs, Tukey post hoc analyses are also reported (p-values and effect sizes, *d*).

|  | **ANOVA** | | | **Tukey Post Hoc** | | | | | |
| --- | --- | --- | --- | --- | --- | --- | --- | --- | --- |
|  | **F-Statistic** | **p-value** | $\boldsymbol{\eta}^{\boldsymbol{2}}$ | **0%-15%** | | **0%-30%** | | **15%-30%** | |
|  |  |  |  | **p-value** | ***d*** | **p-value** | ***d*** | **p-value** | ***d*** |
| *Obstacle Time (s) ˠ* | F(1.3,20.0)  = 13.8 | <0.001*^§^* | 0.48 | <0.001*^§^* | 0.71 | <0.001*^§^* | 1.65 | 0.30 | 0.46 |
| *Distance Traveled (m)* | F(1.3,19.3)  = 3.0 | 0.09 | 0.17 | - | - | - | - | - | - |
| *Avg. Body Speed (m/s)* | F(2,30)  = 7.9 | <0.01*^†^* | 0.35 | 0.15 | 0.31 | <0.01*^†^* | 0.80 | 0.12 | 0.48 |
| *Avg. Turn Radius (m)* | F(2,30)  = 1.8 | 0.19 | 0.11 | - | - | - | - | - | - |
| *Avg. Turn Speed (m/s)* | F(2,30)  = 1.3 | 0.29 | 0.08 | - | - | - | - | - | - |
| *Avg. Max. Turn Speed (m/s)* | F(2,30)  = 1.9 | 0.17 | 0.11 | - | - | - | - | - | - |
| *Avg. Straight. Speed (m/s)* | F(2,30)  = 9.2 | <0.001*^§^* | 0.38 | 0.17 | 0.30 | <0.01*^†^* | 0.87 | 0.07 | 0.53 |
| *Avg. Max. Straight. Speed (m/s)* | F(2,30)  = 14.1 | <0.001*^§^* | 0.48 | 0.08 | 0.32 | <0.001*^§^* | 0.95 | 0.02* | 0.56 |
| *Acceleration Range (m/s^2^)* | F(2,30)  = 15.1 | <0.001*^§^* | 0.50 | 0.06 | 0.33 | <0.001*^§^* | 0.86 | 0.02* | 0.46 |

*Significant at α = 0.05*, 0.01^†^, 0.001^§^*

*ˠ Inverse Transform*

*
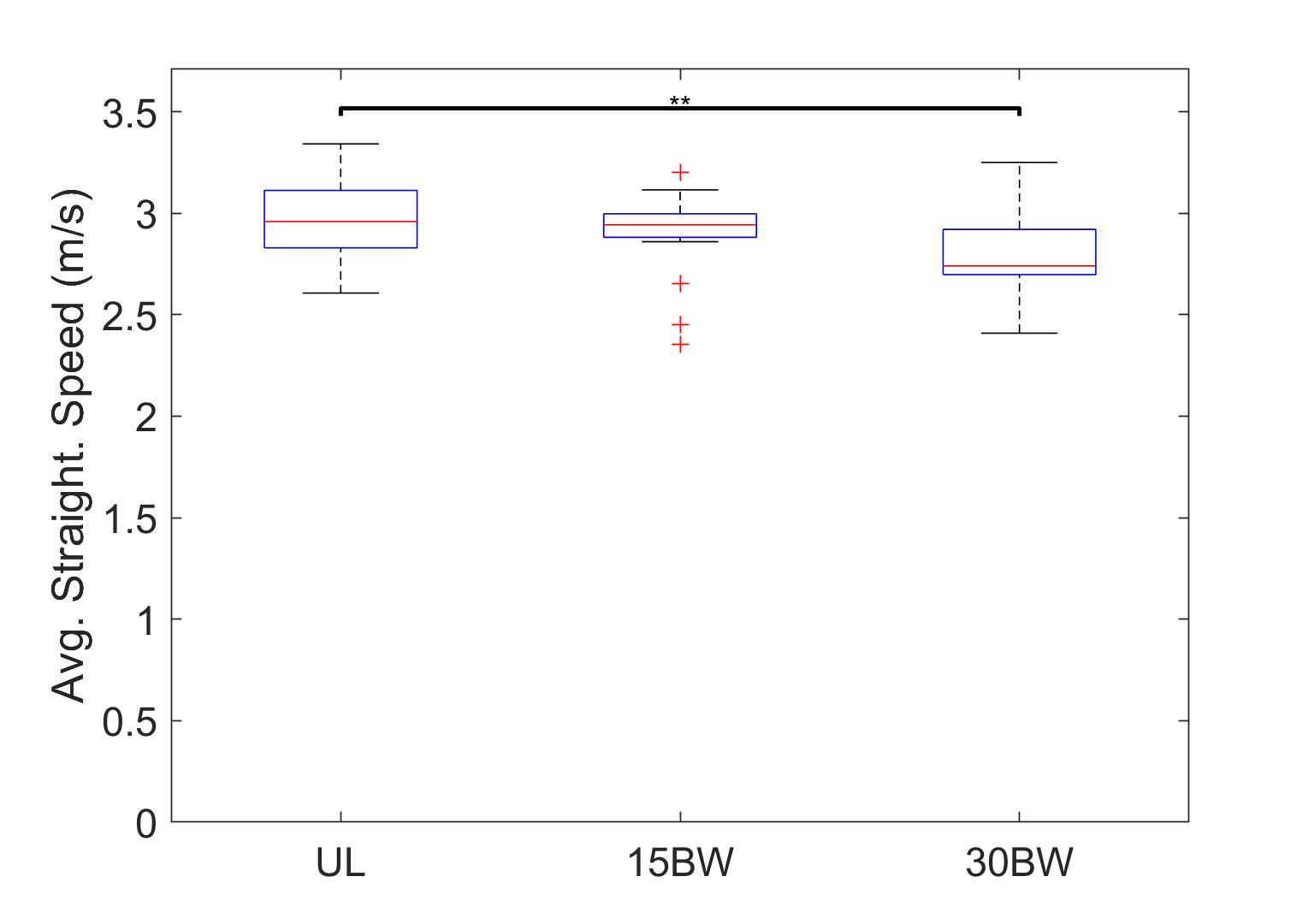
*

**Fig C. Additional agility run statistical results.** Boxplot depicting the results from the Tukey post hoc analysis for the average straightaway speed. The bars denote significant differences between loading conditions at a significance level α = 0.05*, 0.01**, 0.001***

*Bounding Rush*

**Table H. Bounding rush statistical results.** Results from the repeated-measures ANOVA (F-statistics with degrees of freedom, p-values, and effect sizes, $\eta^{2}$). For performance metrics with significant ANOVAs, Tukey post hoc analyses are also reported (p-values and effect sizes, *d*).

|  | **ANOVA** | | | **Tukey Post Hoc** | | | | | |
| --- | --- | --- | --- | --- | --- | --- | --- | --- | --- |
|  | **F-Statistic** | **p-value** | $\boldsymbol{\eta}^{\boldsymbol{2}}$ | **0%-15%** | | **0%-30%** | | **15%-30%** | |
|  |  |  |  | **p-value** | ***d*** | **p-value** | ***d*** | **p-value** | ***d*** |
| *Rushing Speed (m/s)* | F(1.4,18.3)  = 22.2 | <0.001*^§^* | 0.25 | <0.01*^†^* | 0.63 | <0.001*^§^* | 1.10 | <0.01*^†^* | 0.46 |
| *Dropping Speed (m/s)* | F(2,34)  = 7.56 | <0.01*^†^* | 0.31 | 0.08 | 0.24 | 0.02* | 0.50 | 0.28 | 0.20 |
| *Standing Speed (m/s)* | F(2,34)  = 16.5 | <0.001*^§^* | 0.49 | 0.04* | 0.48 | <0.001*^§^* | 1.09 | 0.02* | 0.66 |
| *Sprinting Speed (m/s)* | F(2,34)  = 15.3 | <0.001*^§^* | 0.47 | <0.01*^†^* | 0.56 | <0.001*^§^* | 0.90 | 0.06 | 0.33 |
| *Avg. Dropping Power (W)* | F(1.4,23.7)  = 0.33 | 0.64 | 0.02 | - | - | - | - | - | - |
| *Avg. Standing Power (W)* | F(2,34)  = 1.67 | 0.20 | 0.09 | - | - | - | - | - | - |

*Significant at α = 0.05*, 0.01^†^, 0.001^§^*

*High Crawl*

**Table I. High crawl statistical results.** Results from the repeated-measures ANOVA (F-statistics with degrees of freedom, p-values, and effect sizes, $\eta^{2}$). For performance metrics with significant ANOVAs, Tukey post hoc analyses are also reported (p-values and effect sizes, *d*).

|  | **ANOVA** | | | **Tukey Post Hoc** | | | | | |
| --- | --- | --- | --- | --- | --- | --- | --- | --- | --- |
|  | **F-Statistic** | **p-value** | $\boldsymbol{\eta}^{\boldsymbol{2}}$ | **0%-15%** | | **0%-30%** | | **15%-30%** | |
|  |  |  |  | **p-value** | ***d*** | **p-value** | ***d*** | **p-value** | ***d*** |
| *Crawl Speed (m/s)* | F(1.4,18.5)  = 43.2 | <0.001*^§^* | 0.77 | <0.01*^†^* | 0.55 | <0.001*^§^* | 1.12 | <0.01*^†^* | 0.57 |
| *Crawl Stride Time (s)* | F(2,26)  = 18.1 | <0.001*^§^* | 0.58 | <0.01*^†^* | -0.68 | <0.01*^†^* | -0.98 | 0.03* | -0.39 |
| *Contralateral Coordination* | F(2,26)  = 9.44 | <0.001*^§^* | 0.42 | 0.27 | 0.28 | <0.01*^†^* | 0.81 | 0.04* | 0.49 |
| *Ipsilateral Coordination* | F(2,26)  = 4.25 | 0.03* | 0.25 | 0.31 | -0.32 | 0.07 | -0.67 | 0.23 | -0.33 |

*Significant at α = 0.05*, 0.01^†^, 0.001^§^*

*Vertical Transfer*

**Table J.** **Vertical transfer obstacle statistical results.** Results from the repeated-measures ANOVA (F-statistics with degrees of freedom, p-values, and effect sizes, $\eta^{2}$). For performance metrics with significant ANOVAs, Tukey post hoc analyses are also reported (p-values and effect sizes, *d*).

|  | **ANOVA** | | | **Tukey Post Hoc** | | | | | |
| --- | --- | --- | --- | --- | --- | --- | --- | --- | --- |
|  | **F-Statistic** | **p-value** | $\boldsymbol{\eta}^{\boldsymbol{2}}$ | **0%-15%** | | **0%-30%** | | **15%-30%** | |
|  |  |  |  | **p-value** | ***d*** | **p-value** | ***d*** | **p-value** | ***d*** |
| *Duration of Lift (s)* | F(2,34) = 1.1 | 0.351 | 0.06 | - | - | - | - | - | - |
| *Duration of Lowering (s)* | F(2,34) = 1.5 | 0.232 | 0.08 | - | - | - | - | - | - |
| *Lift Smoothness* | F(2.0,34.2) = 1.7 | 0.200 | 0.09 | - | - | - | - | - | - |
| *Lowering Smoothness* | F(2,34) = 0.2 | 0.852 | 0.01 | - | - | - | - | - | - |

*Significant at α = 0.05*, 0.01^†^, 0.001^§^*
